# The Role of Canine Distemper Virus and Persistent Organic Pollutants in Mortality Patterns of Caspian Seals (*Pusa caspica*)

**DOI:** 10.1371/journal.pone.0099265

**Published:** 2014-07-02

**Authors:** Susan C. Wilson, Tariel M. Eybatov, Masao Amano, Paul D. Jepson, Simon J. Goodman

**Affiliations:** 1 Tara Seal Research Centre, Killyleagh, County Down, Northern Ireland, United Kingdom; 2 Institute of Geology, Baku, Azerbaijan; 3 Faculty of Fisheries, Nagasaki University, Nagasaki, Japan; 4 Institute of Zoology, Zoological Society of London, London, United Kingdom; 5 School of Biology, University of Leeds, Leeds, United Kingdom; NIH, United States of America

## Abstract

Persistent organic pollutants are a concern for species occupying high trophic levels since they can cause immunosuppression and impair reproduction. Mass mortalities due to canine distemper virus (CDV) occurred in Caspian seals (*Pusa caspica*), in spring of 1997, 2000 and 2001, but the potential role of organochlorine exposure in these epizootics remains undetermined. Here we integrate Caspian seal mortality data spanning 1971–2008, with data on age, body condition, pathology and blubber organochlorine concentration for carcases stranded between 1997 and 2002. We test the hypothesis that summed PCB and DDT concentrations contributed to CDV associated mortality during epizootics. We show that age is the primary factor explaining variation in blubber organochlorine concentrations, and that organochlorine burden, age, sex, and body condition do not account for CDV infection status (positive/negative) of animals dying in epizootics. Most animals (57%, n = 67) had PCB concentrations below proposed thresholds for toxic effects in marine mammals (17 µg/g lipid weight), and only 3 of 67 animals had predicted TEQ values exceeding levels seen to be associated with immune suppression in harbour seals (200 pg/g lipid weight). Mean organonchlorine levels were higher in CDV-negative animals indicating that organochlorines did not contribute significantly to CDV mortality in epizootics. Mortality monitoring in Azerbaijan 1971–2008 revealed bi-annual stranding peaks in late spring, following the annual moult and during autumn migrations northwards. Mortality peaks comparable to epizootic years were also recorded in the 1970s–1980s, consistent with previous undocumented CDV outbreaks. Gompertz growth curves show that Caspian seals achieve an asymptotic standard body length of 126–129 cm (n = 111). Males may continue to grow slowly throughout life. Mortality during epizootics may exceed the potential biological removal level (PBR) for the population, but the low frequency of epizootics suggest they are of secondary importance compared to anthropogenic sources of mortality such as fishing by-catch.

## Introduction

Marine mammals are frequently cited as sentinels for marine ecosystem health and function. In particular there is interest in their role as indicators for direct toxic impacts of bioaccumulative persistent organic pollutants (POPs) such as organochlorines (OCs) [1]–[8] and how environmental contaminants may modulate responses to natural stressors, such as infectious disease and food availability [9]. Between 1997 and 2001, Caspian seals (*Pusa caspica*) suffered a series of mass mortalities, affecting 1000 s of animals, which raised international concern both about the status of the Caspian seal as a species and the broader Caspian Sea ecosystem [10], [11]. Investigation of the mass mortalities determined Canine Distemper Virus (CDV) as the cause [11]–[13], but concern remains over the potential role in the epizootics of pollution and other environmental factors [14], [15].

The Caspian seal is a small bodied, ice-breeding phocid seal endemic to the Caspian Sea, which is the largest landlocked water body on the planet. Since the early 20^th^ Century the Caspian Sea has been subject to impacts from invasive species, industrial development, pollution, habitat loss, and unsustainable extraction of natural resources [10]. Against this background of broader ecosystem change, the Caspian seal population has declined by 90% from more than 1 million to around 100,000 individuals since the end of the 19^th^ Century, primarily due to unsustainable commercial hunting [16], [17]. The species is listed as Endangered in the International Union for Conservation of Nature (IUCN) Red List [10].

A previously unknown strain of CDV was identified from a dead seal in the 1997 mortality [12] and initial toxicology results revealed high levels of organochlorine contaminants in blubber [18]. Pathological investigations indicating CDV as the cause of the mass mortality in spring 2000 were first reported by Kennedy et al. [13]. Data on organochlorine pesticides (such as dichlorodiphenyltrichloroethanes (DDTs)) and a suite of 25 chlorobiphenyl congeners and trace metals were reported by Kajiwara et al. [14] and Anan et al. [19]. A more detailed study on all aspects of the 2000 mortality was later assembled by Kuiken et al. [11]. Kajiwara et al. [15] collected further OC data from fish and seals dying in autumn 2000 and spring 2001.

Although the Caspian is a major oil producing region, hydrocarbon pollution appears to be an unlikely contributory factor, since levels were found to be undetectable in adult seals in 1997 [20]. Both seals and fish are capable of metabolising hydrocarbons [21]. Metabolic indices indicate a relatively high degradation capacity for hydrocarbons in Caspian seals [22] and this might reflect this species’ history of living in waters contaminated by oil through natural seepage over evolutionary timescales [23]. Trace metals in animals from the 2000 mortality were not significantly elevated, with the exception of Zn and Fe in some animals, which were attributed to a metabolic disorder and redistribution of trace metals in diseased animals [4], [19].

Kajiwara et al. [14], [15] proposed that OCs consumed by seals eating contaminated fish may compromise immune system function, making them more susceptible to disease. Kajiwara et al. [14], [15] also suggested that OCs levels in Caspian seals were high enough to impair fertility, as has been suggested for harbour seals (*Phoca vitulina*) in the Wadden Sea [5], [6] and ringed seals (*Pusa hispida*) in the Baltic [2], [24]. However, Kuiken et al. [11] concluded that from the samples collected in 2000 there was no evidence for OC involvement in mortality from CDV, although sample sizes were limited.

Resolving the likely impact of OC exposure on Caspian seal health may help to prioritise conservation action. In this paper we integrate records on stranding mortality for Caspian seals dating over 40 years, new age determination data and estimation of Caspian seal growth curves, with data on organochlorine concentrations from animals sampled during the mass mortalities, controlling for confounding factors not considered in previous studies such as age, sex and nutritive status. We revaluate the relative importance of organochlorine (polychlorinated biphenyls (PCBs) and DDTs) pollutant exposure as contributory factors to the Caspian CDV epizootics, and assess the impact of epizootics against long term mortality patterns and current anthropogenic caused mortality.

## Methods

### Long term mortality monitoring and sampling

In Azerbaijan, systematic monitoring of the coast for carcases was carried out monthly between 1971 and 1989 (with the exception of 1979) and between 1997 and 2008. The monitoring was conducted on a fixed 10 km stretch of beach on the north side of the Apsheron Peninsula, between ∼40.523 N, 50.119 E and 40.501 N, 50.226 E (Figure S1 in File S1). The beach was driven or walked at least once a month and newly stranded carcases were measured, and the sex of the seal recorded. Each carcase was cut along the chest to identify it as having been examined.

Standard body length was measured as straight length from tip of nose to tip of tail with the exception of carcases in Iran, for which curvilinear body length was measured; the carcases from Iran are therefore omitted from analyses involving body length. Blubber thickness was measured in the mid-ventral location (between fore flippers).

Tissue samples for pathology and toxicology analysis were collected from a total of 67 Caspian seal carcases found dead on the Caspian shoreline in Azerbaijan, Iran, Kazakhstan, Russian Federation, and Turkmenistan (Figure S1 and Table S1 in File S1) during the 1997–2002 mortality investigations. Samples were collected as described in Kennedy et al. [13], and Kuiken et al. [11]. The pathology data analysed here include results from 14 of 18 carcases necropsied and analysed in detail by Kuiken et al. [11] plus an additional 25 carcases from which samples for virology were taken between August to December 2000, April to September 2001 and March 2002 (Table S1 in File S1), results for which have not been previously reported. The toxicology data have been previously published [14], [15], but are reanalysed here in the context of new age and pathology data. Some supporting data, e.g. body length, age, blubber thickness and CDV status, were not available for all individuals with toxicology data. Samples sizes for individual statistical analyses are indicated as appropriate.

### Age determination

Where the condition of carcases allowed, a canine tooth was taken for age determination from seals examined during the 1997–2002 mortalities. Age was determined as described by Amano et al. [25] using decalcified and haematoxylin-stained canine teeth. For the 1997 carcases, cross-sections were made from an upper canine tooth [26], while for the 2000–02 specimens, both cross and longitudinal sections were made from a lower canine tooth [25]. In both cases, growth layers were counted in both dentine and cementum.

### Pathology and Virology

Necropsies and morbillivirus virological analyses of freshly dead carcases found in May–June 2000–2001 (Table S1 in File S1) were conducted using standardised protocols [11], [13]. Tissue samples were tested for presence of morbillivirus antigen by an immunohistochemical (IHC) technique and for morbillivirus nucleic acid by reverse-transcriptase polymerase chain reaction (RT-PCR). RT-PCR assays were later carried out on samples from late summer and winter 2000 and spring 2001, although most carcases from these periods were in varying states of decomposition.

### Toxicology

Blubber samples were taken from the mid-ventrum of each carcase and wrapped in foil. The material was transported in liquid nitrogen dry-shippers and archived at −80°C prior to analysis. Samples were analysed by Kajiwara et al. [14], [15] for a range of organochlorine contaminants, including residues from industrial lubricants, PCBs and pesticide residues (DDTs), and HCHs (hexachlorocyclohexanes). The 25 chlorobiphenyl congeners (CBs) analysed included all o*rtho*-substituted congeners tri-to-octa-CBs and non-*ortho* coplanar congeners (IUPAC 77, 126 and 169; [14], [15]). The concentrations were analysed using an equivalent mixture of Kanechlor preparations with known PCB composition and content [14], [15], [27]. The concentrations of DDTs and other organochlorines, including HCHs, were quantified using internationally standardised methodologies [28]. For the present analysis the ΣPCBs and ΣDDTs (measured as µg/g lipid weight) are used as an indicator of contaminant levels in seal blubber.

### Data analysis

All data analysis was conducted using the R statistical package [29]. Potential periodicity in the long term Apsheron long term annual mortality time series was tested by fitting a chi-square periodgram [30] in *R*.

Age estimates derived from teeth and standard body length measurements were used to derive growth curves for males and females separately, and for the total data, using a single parameter Gompertz growth model. Gompertz growth models have been used widely in past studies of marine mammal growth [31]–[34]. A single parameter model was chosen due to the relatively small sample sizes across age ranges when partitioning by sex, rather than using multi-parameter models, such as a double-Gompertz, which could identify different growth phases given sufficient data. Age and length at physical maturity were predicted from a growth curve given by:
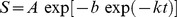
where *S* is a measure of body length, *A* is the asymptotic body length value, *b* is an integration constant, *k* is the growth rate constant and *t* is the tooth-based age [35]–[37]. Parameters *A*, *b*, and *k*, with associated standard errors were estimated from the age and length data via non linear least-squares methods using the *nls* function in *R* [29]. Fitted models for the combined dataset and a nested model with parameters estimated separately for males and females were compared using Akaike Information Criterion (AIC) scores, and Akaike evidence ratio. In addition 95% confidence intervals for the estimated parameters and fitted growth curves were estimated through bootstrapping.

For the toxicology data, numerical variables were assessed for conformation to a normal distribution and transformed where appropriate. Explanatory variables contributing to variation in toxicology measures were assessed by fitting linear models. Minimum adequate models were determined through parameter addition and removal. Models were compared using AIC evidence ratios and analyses of variance. Contributions of explanatory variables to the CDV status of individuals (positive or negative for CDV infection) were assessed via Welch two-sample *t*-tests, and logistic regression with general linear binomial models with logit-link function.

### Research Ethics

No animals were killed for the purposes of this study. All investigations were conducted using material collected from individuals which died of natural causes. No special ethical approval was therefore required for this work.

## Results

### Mortality monitoring on the Apsheron Peninsula

**Figure 1:**
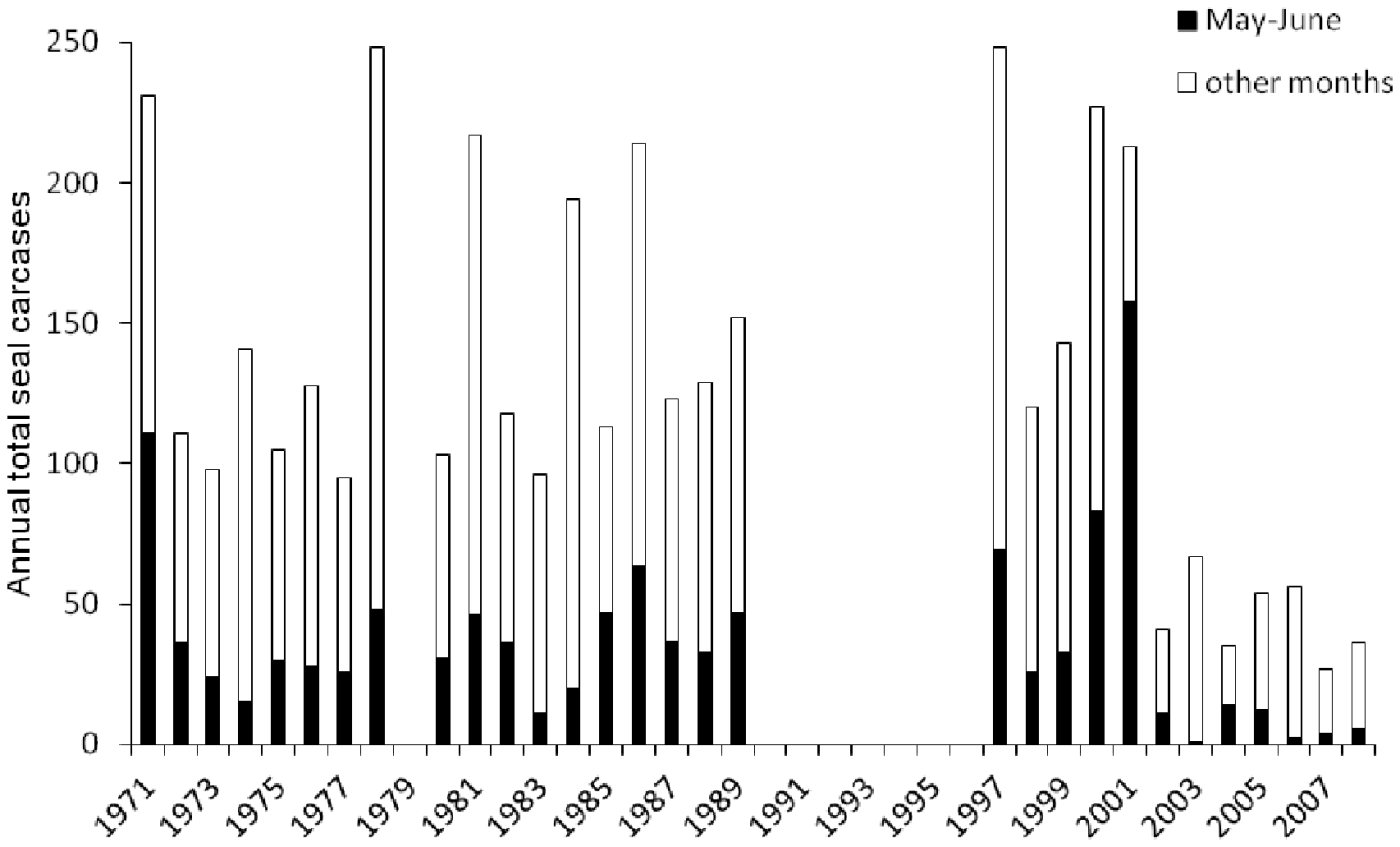
Time series histogram for annual Caspian seal mortality 1971–2008, along the 10 km Apsheron monitoring zone (40.523 N, 50.119 E and 40.501 N, 50.226 E).

**Figure 2:**
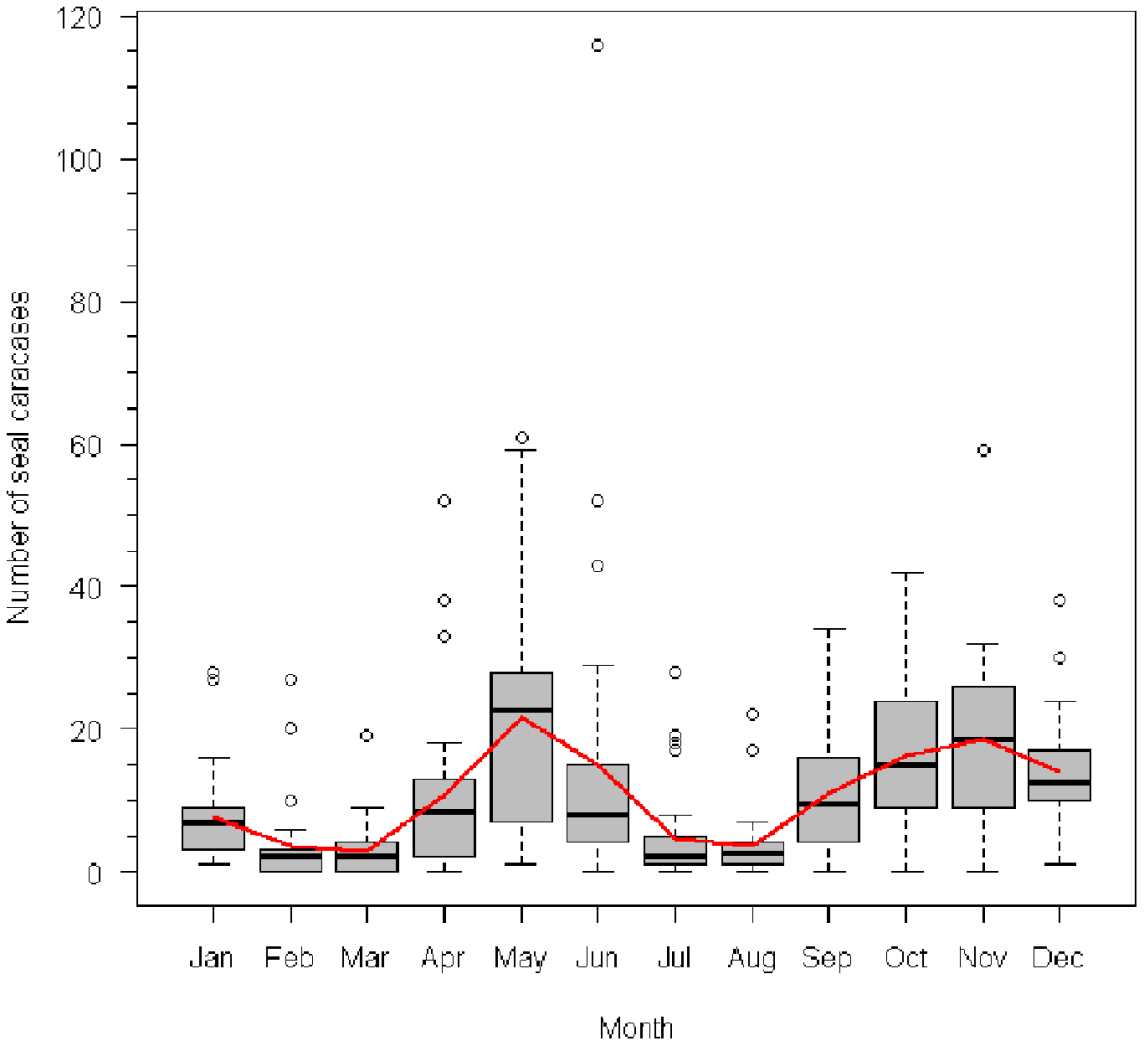
Boxplot for number of Caspian seal carcases recorded monthly (1971–2008) in the 10 km Apsheron monitoring zone. Central line of boxes gives median carcases per month. Red line gives mean carcase number.

The mean annual carcase count 1971–1990 was 145.33, falling to 45.14 (Wilcoxon rank sum, *W* = 126, *P* = 4.16e^−06^) 2002–2008 after the epizootic years (Figure 1). There were five years between 1971 and 1989 with higher counts, as well as the epizootic years, 1997, 2000 and 2001, when the number of carcases was 200–250 (Figure 1). Peaks of carcase strandings are observed in the spring (April-June), and in late autumn (September-December; Figure 2). The average number of carcases found in May–June 1971–1989 was 38, but exceeded 100 in 1971 and 2001, and 50 in 1986, 1997 and 2000 (Figures 1, 2). We tested for evidence of periodicity in the mortality time series using a chi-square periodgram analysis. This yielded a suggestive, but non-significant, signal of a 7 year period (*P* = 0.061) for mortality in the spring (May-June) when the most recent epizootics occurred. For overall annual mortality, a period of 3 years was returned, but again this was non-significant (*P* = 0.081).

### Relationship between age and body length

**Figure 3:**
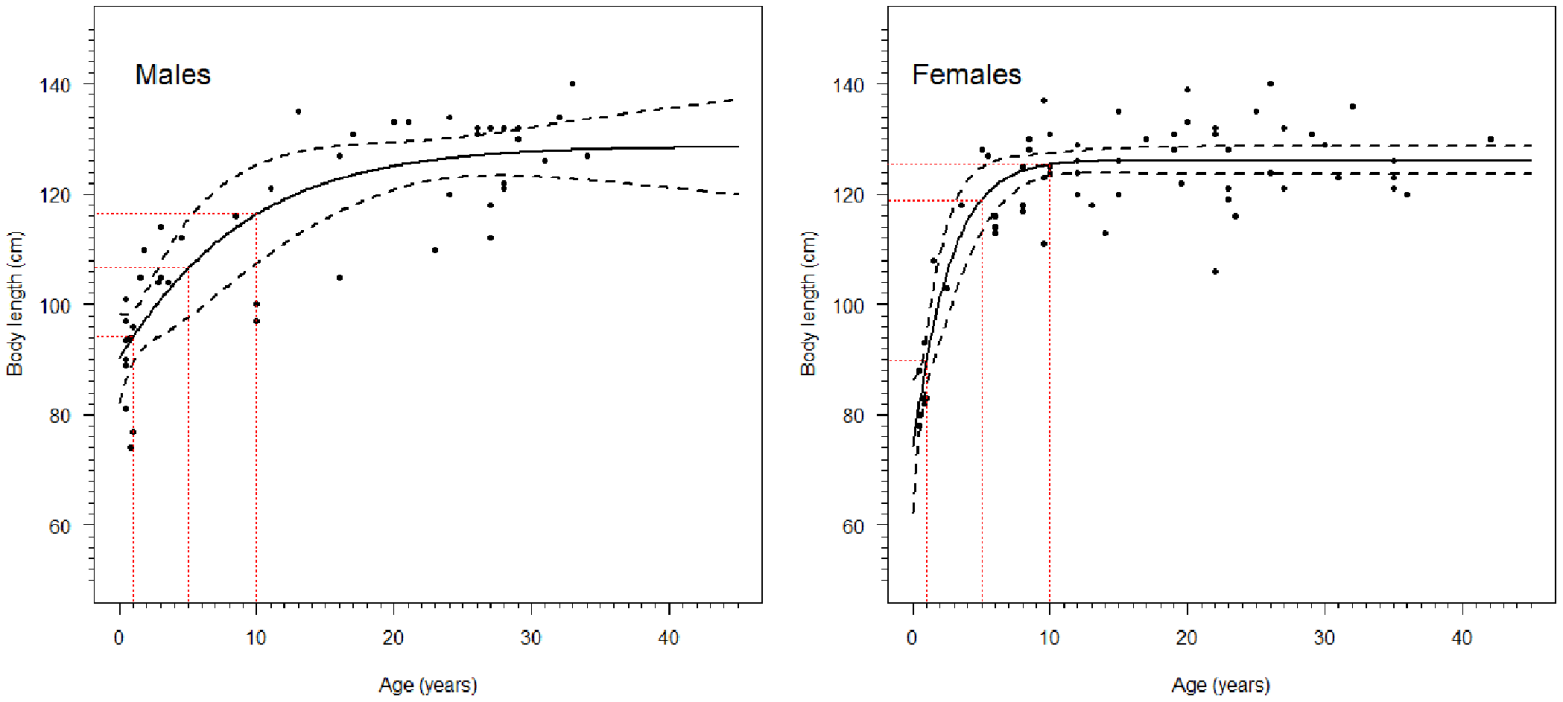
Single parameter Gompertz growth curves for male (left) and female (right) Caspian seals. Points are observed data, solid black line fitted growth curve, dashed black line are bootstrap derived 95% confidence intervals around the fitted curve, red dashed lines indicate points on the curve at 1, 5 and 10 years of age.

Tooth-based age and standard body length measurements were available for a total of 111 individuals, comprising 45 males and 66 females. Ages ranged from 6 months to 44 years (Figure 3; Table S2 and Figure S2 in File S1). The nested model with separate parameter estimates for males and females yielded a significantly better fit than a single growth curve fitted to the combined data for both sexes (ΔAIC −3.58, Evidence ratio 1∶5.989). Predicted asymptotic lengths for males and females in the nested model were ∼129 cm and ∼126 cm respectively (Table 1) but there was considerable variation around these values in the actual data with a maximum recorded length of 140 cm (Figure 3). Comparison of bootstrap 95% confidence intervals shows there is no significant difference in the asymptotic length between sexes, and there was a much higher standard deviation for males compared to females. The fitted growth curves suggest males are initially larger than females early in the first year of life ∼90 cm compared to ∼80 cm, but females have more rapid initial growth rates and start to overtake males in their second year, reaching ∼119 cm by 5 years compared to ∼107 cm for males (Figure 3). Females appear to reach their asymptotic length after 10 years, while males exhibit almost continuous, slower growth until beyond the age of 30 (Figures 3, S2 in File S1). However, given the relatively high standard deviation in lengths for older age classes these differences in growth curves should be treated with some caution.

**Table 1:** Comparison of Gompertz growth model parameters nested by Sex.

Sex	Parameter	Estimate	nls S.E	t	Pr(>|t|)	Bootstrap S.E	Lower 95% CI	Upper 95% CI
Male	*A*	128.656	2.942	43.724	<2e^−16^	9.039	110.938	146.373
	*k*	0.127	0.039	3.194	0.0018	0.081	−0.032	0.284
	*b*	0.355	0.031	11.583	<2e^−16^	0.054	0.249	0.459
Female	*A*	126.157	1.250	100.895	<2e^−16^	1.281	123.646	128.668
	*k*	0.440	0.106	4.162	6.48e^−05^	0.180	0.086	0.793
	*b*	0.529	0.066	8.072	1.22e^−12^	0.087	0.358	0.699

*A* – asymptotic length, *b* – integration constant, *k* – growth rate constant, nls S.E – standard error derived from nonlinear least squares model, Bootstrap S.E – Standard error derived from 1000 bootstrap resamplings of the data and refitting of the model.

### Age structure of mortality 1997–2002

**Figure 4:**
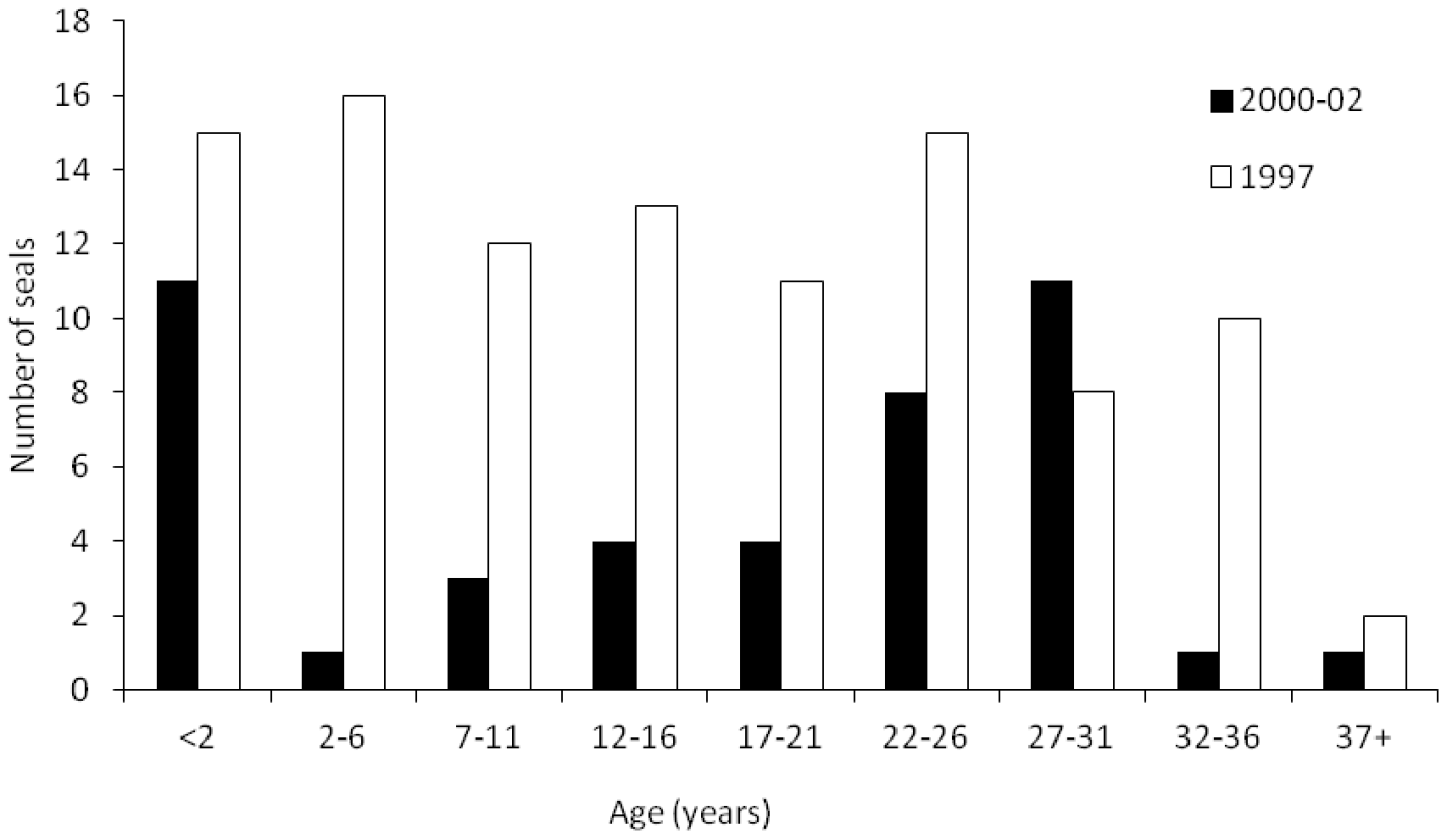
Histogram of age structure among Caspian seal carcases stranded 1999–2002.

Ages were determined for 102 dead seals from 1997 and 44 dead seals from 2000–02 (Figure 4). In 2000–02 the majority of dead seals were either less than 2 years or greater than 22 years of age, with fewer carcases than expected in the 2–11 age range (χ^2^ = 14.26; d.f. = 2; P<0.01, comparing age groups 2–11, 12–21 and >22). The age distribution in these three age classes differed significantly between 2000–02 and 1997 (χ^2^ = 6.41; d.f. = 2; P<0.05). In 1997 the distribution of dead seals in these three age groups was not significantly different (χ^2^ = 3.13; d.f. = 2; P>0.30).

### Toxicology of seal carcases 1997–2002

**Figure 5:**
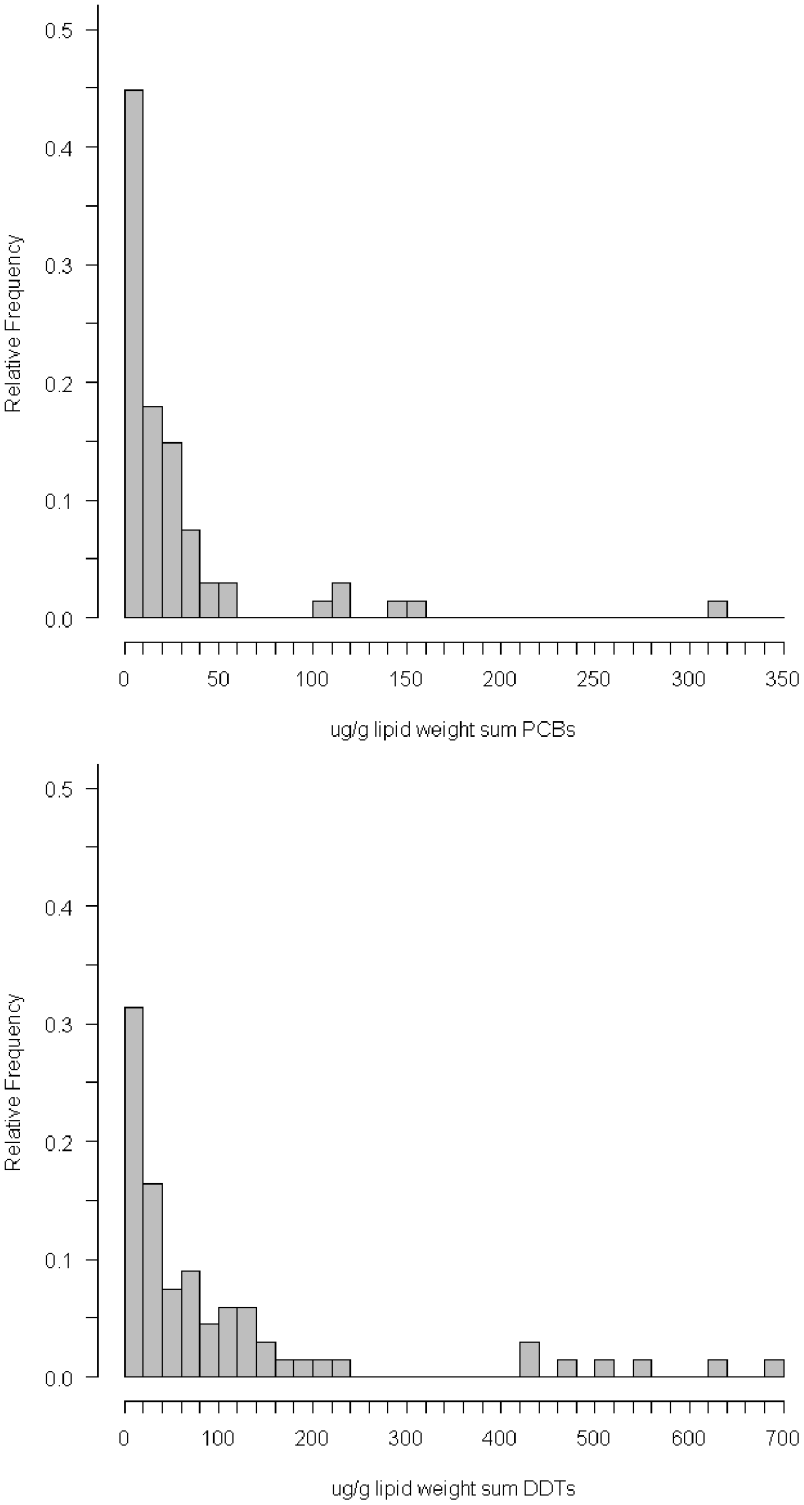
Relative frequency histograms showing distribution of ∑PCB and ∑DDT levels for necropsied seals (n = 67).

**Figure 6:**
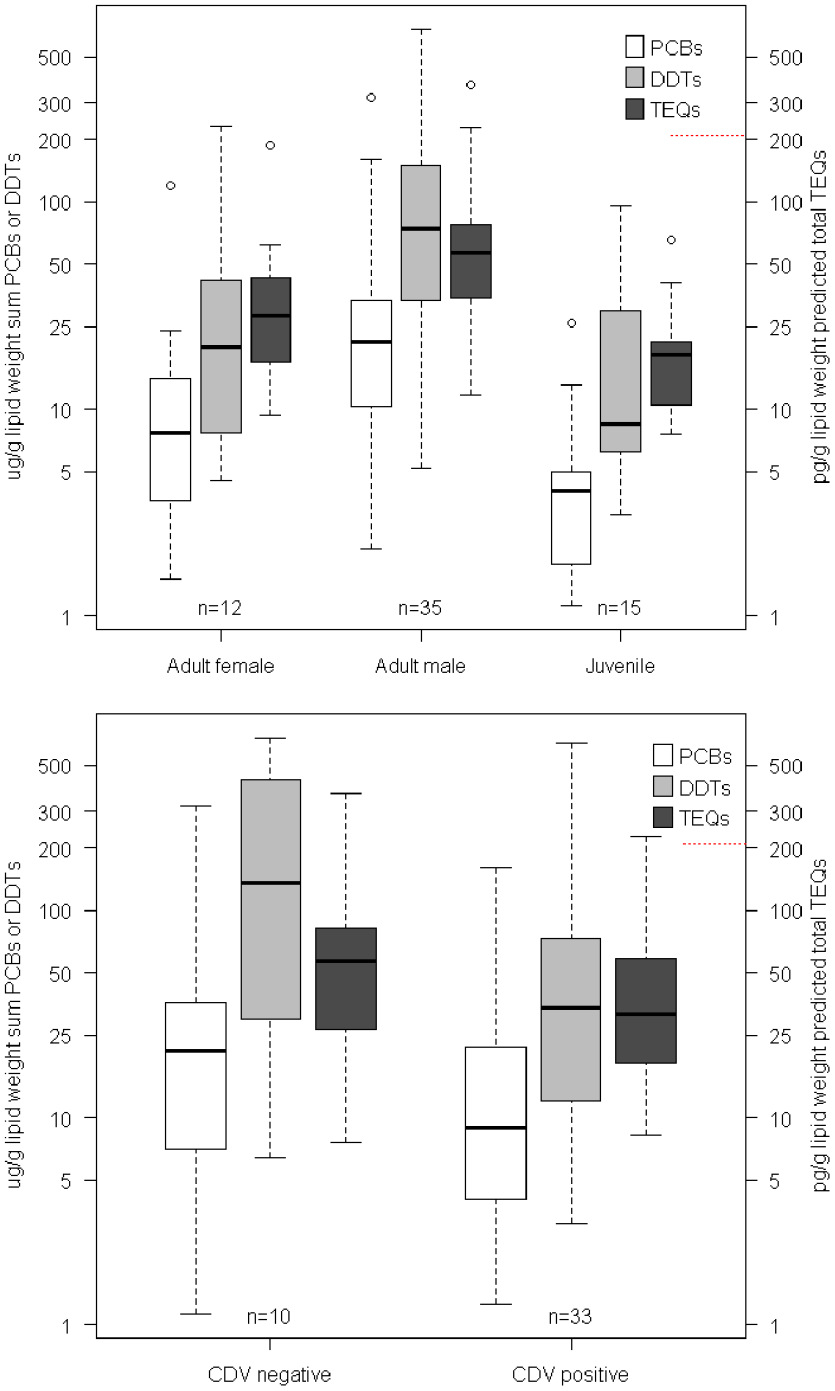
Boxplots of contaminant burdens by sex-age class (top) and CDV status (bottom). Red dashed line indicates proposed threshold for potential toxic effects for ∑TEQ levels (196−220 pg/g lipid weight) derived from experimental studies in harbour seals (De Swart et al. 1994, 1995a, 1995b, 1996, Ross et al. 1995). y axis is plotted on log10 scale.

Mean ∑PCB concentrations (n = 67) were 28.599 µg/g lipid weight, range 1.12–320 µg/g lipid weight, and mean ∑DDTs (n = 67) were 108.927 µg/g lipid weight, range, 3.1–684 µg/g lipid weight (Figure 5). Blubber ∑PCB concentrations were highest in adult males (n = 35; mean = 34.996 µg/g lipid weight), moderately high in adult females (n = 12; mean = 17.827 µg/g lipid weight) and lowest in juveniles (n = 15; mean = 5.491 µg/g lipid weight) (Figure 6). ∑DDTs concentrations were highest in adult males (n = 35; mean = 145.774 µg/g lipid weight), moderately high in adult females (n = 12; mean = 41.308 µg/g lipid weight) and lowest in juveniles (n = 15; mean = 25.755 µg/g lipid weight) (Figure 6).

∑PCB and ∑DDT concentrations, age, and blubber thickness (hereafter referred to as Blubber) were assessed for conformation to a normal distribution using a Shapiro-Wilk’s normality test. All variables showed a significant deviation from normality (data not shown). Log10 transformed values for ∑PCB and ∑DDT concentrations did fit a normal distribution, but not Age or Blubber+1 (data not shown), although the frequency histogram for log10(Blubber+1) did approximate a normal distribution. All further analysis was done with the log10 transformed values except for Age.

Log10 PCBs varied significantly among Sex-age classes (Adult males, Adult females, Juveniles), and Countries, while log10 DDTs varied significantly among Sex-age classes, Countries and Season (Table 2, Figure 6, Figure S3 in File S1). Log10(Blubber+1) varied significantly among Countries and Season, but not among Sex-age classes or Years (Table 2, Figure S4 in File S1). The was no significant association between log10(Blubber+1) and Age for either males (Adjusted *R* ^2^: −0.03148, F_1,29_∶0.0844, *p* = 0.7735) or females (Adjusted *R* ^2^∶0.024, F_1,11_∶1.295, p = 0.279) (see Figure S4 in File S1). The variation in Season is likely to be conflated with Country, since all Autumn samples were collected in Iran, while Spring samples came from Azerbaijan, Kazakhstan and Turkmenistan.

**Table 2:** Anova comparison for log10(PCBs), log10(DDTs) and log10(Blubber+1) versus, Sex-age class, Country, Year and Season.

	Comparison	*F*	df	*P*
**PCBs**	Sex-age class	12.24	2	3.57e^−05^
	Country	4.837	4	0.0018
	Year	1.179	1	0.2820
	Season	3.719	1	0.0582
**DDT**	Sex-age class	12.63	2	2.72e^−05^
	Country	5.641	4	0.0006
	Year	0.396	1	0.5320
	Season	4.422	1	0.0393
**Blubber**	Sex-age class	0.344	2	0.7110
	Country	2.402	4	0.0775
	Year	0.038	1	0.8470
	Season	5.153	1	0.0270

The contributions of Age, Sex, and Blubber thickness to variation in log10 ∑PCB and ∑DDT concentrations were assessed by fitting linear models. Initially Country was also included as term, and did appear to explain a significant proportion of the variation of in OC burdens. However, since Caspian seals form a single population which migrates throughout the Caspian, there is no mechanism to drive spatial variation in OC exposure. Moreover, due to the relative small and unbalanced sample sizes, Country and Season are likely to conflate variation represented in Sex, Age, Blubber thickness, hence the former terms were omitted to avoid redundancy and over parameterisation of the models.

Age, Sex and log10(Blubber+1) all explained significant variation in both PCBs and DDTs when fitted singularly (Tables S3, S4 in File S1). PCBs and DDTs were positively correlated with Age, and negatively correlated with log10(Blubber+1) in adults, but not juveniles (see Figure S3 and S5 in File S1). Models with multiple explanatory variables yielded significantly better fits in each case. For both PCBs and DDTs the models with the lowest (best) AIC scores included Age, Sex, and an interaction term for Age and log10(Blubber+1) (Tables S3, S4 and Figure S5 in File S1).

In the PCB models, there was strong support across all models for Age explaining a significant proportion of the variance. There was less consistency for log10(Blubber+1), Sex, and interaction terms, despite being present in the top ranked models on the basis of AIC, with only the Age:log10(Blubber+1) interaction, showing marginal significance in some models. This suggests the PCB data lacks sufficient power to be able to attribute sources of variation unambiguously. In models including all 3 main terms, Age accounted for ∼7 times more variance than log10(Blubber+1), and ∼20 times more than Sex (Table 3).

**Table 3:** Anova tables for highest ranked PCB and DDT models containing the 3 main terms Age, log10(Blubber+1), and Sex.

log10(PCBs)∼Age+log10(Blubber+1)+Age:log10(Blubber+1)+Sex:log10(Blubber+1)+Sex						AIC = 60.41645
	Df	Sum Sq	Mean Sq	*F* value	*Pr*(>*F*)	
Age	1	3.725	3.725	19.135	9.16e^−05^	***
log10(Blubber+1)	1	0.513	0.513	2.633	0.1129	
Sex	1	0.188	0.188	0.967	0.3317	
Age:log10(Blubber+1)	1	0.8	0.8	4.111	0.0497	*
log10(Blubber+1): Sex	1	0.42	0.42	2.157	0.1502	
Residuals	38	7.398	0.195			
**log10(DDTs)∼Age+log10(Blubber+1)+Age:log10(Blubber+1)+Sex)**						**AIC = 60.74518**
	**Df**	**Sum Sq**	**Mean Sq**	***F*** ** value**	***Pr*** **(>** ***F*** **)**	
Age	1	2.823	2.8228	14.113	0.0006	***
log10(Blubber+1)	1	1.188	1.1883	5.941	0.0195	*
Sex	1	1.108	1.1084	5.542	0.0237	*
Age:log10(Blubber+1)	1	0.825	0.8247	4.123	0.0492	*
Residuals	39	7.8	0.2			

*P<0.05, ***P<0.001.

Age, Sex, and the Age:log10(Blubber+1) interaction, were consistently supported as significant terms in the top ranked DDT models. Log10(Blubber+1) appeared as a significant explanatory variable from the 3^rd^ ranked model onwards, but the slope associated with this term was not significantly different from zero in any model (data not shown). As with PCBs, this suggests there may be lack of power in the data to fully resolve contributions to the variance in DDTs. Age accounted for ∼2.4 and ∼2.5 times more variance than log10(Blubber+1) and Sex respectively in models including all 3 main terms (Table 3).

Potential impacts of OC burdens on CDV status were tested by comparing PCB and DDT levels in animals confirmed as CDV+ and CDV− through RT-PCR or immunohistochemistry. The CDV− individuals were diagnosed either as having died as a result of bacterial infections ([11], G. Boseret and S. Wilson, unpublished data) or having undetermined causes of death. Therefore it was not possible to perform a broader case-control comparison using individuals partitioned into infectious disease and non-infectious disease categories. Overall, burdens appeared to be higher in CDV− individuals for both PCBs and DDTs (Figure 6), but there were no significant differences in PCB, DDT or blubber thickness between CDV+ and CDV− individuals on the basis of Welch two sample t-tests (Table 4). Logistic regression (general linear binomial model with logit link function) also failed to identify any variable from log10 PCB or DDT, Age, log10(Blubber+1) or Sex, which contributed to differences among CDV+ and CDV− animals (Table S5 in File S1).

**Table 4:** Comparison of PCBs, DDTs and Blubber thickness in CDV+ and CDV− animals (Welch 2 sample *t*-test).

log10(PCBs)∼CDV				
Mean CDV−	Mean CDV+	*t*	df	*p*
1.268	0.939	1.340	11.662	0.2057
**log10(DDTs)∼CDV**				
**Mean CDV**−	**Mean CDV+**	***t***	**df**	***p***
2.009	1.499	2.093	12.819	0.05678
**log10(Blubber+1)∼CDV**				
**Mean CDV**−	**Mean CDV+**	***t***	**df**	***p***
0.512	0.681	−1.678	17.902	0.1105

### Seasonal variation in body condition

**Figure 7:**
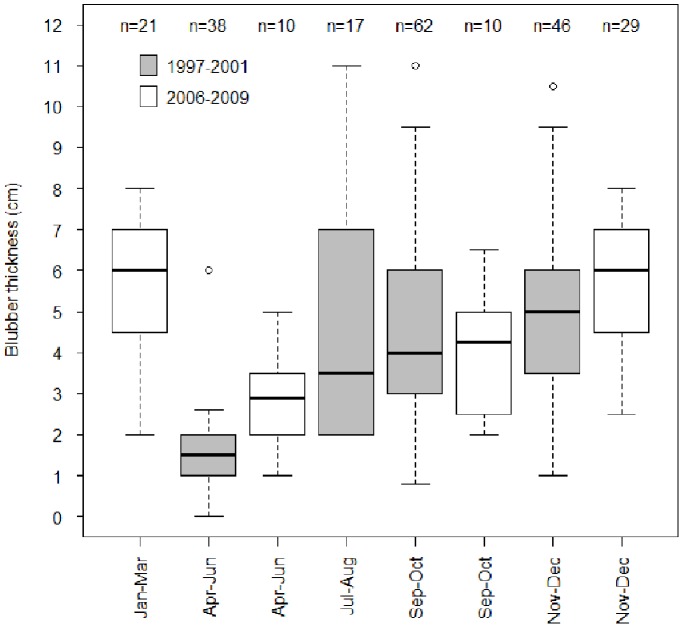
Boxplot of blubber thickness (cm) for Caspian seal carcases stranded 1997–2009, showing seasonal variation in body condition.

Measurements of blubber thickness in stranded carcasses 1997–2009 (n = 233) showed significant seasonal variation (Figure 7), with the lowest blubber thicknesses recorded in late spring-early summer after the breeding-moulting periods (*F* = 35.02, df = 4, *P*<2e^−16^). Blubber thicknesses increased through the summer, peaking in late autumn and winter. Mean blubber thickness was significantly lower in epizootic years (1997, 2000, 2001) than non-epizootic years (2006–2009) (*t* = 4.0097, df = 193.655, *P* = 8.673e^−05^). Mean blubber thickness also appeared lower in epizootic years in each time period where data for comparison was available (Figure 7), but this difference was only significant for the April-June period (*t* = 3.4562, df = 12.697, *P* = 0.0044).

## Discussion

### Long term mortality monitoring

Mortality monitoring of the Apsheron peninsula spanning 37 years identified peaks of mortality in spring and autumn, reflecting seasonal fluctuations in seal numbers. The site lies along the migration corridor used by seals moving south into foraging areas in the southern Caspian after winter breeding and moulting periods, and then returning north in autumn or early winter in preparation for breeding on the northern ice-sheet [38].

Since the identification of CDV in Caspian seals, a key question has been whether the virus was endemic and caused mortalities prior to 1997. Antibodies to CDV have been detected in archive serum from 1993, 1997 and 1998 [39]. Morbilliviruses typically cause epidemics with a periodicity dependent on the build- up of susceptible individuals [40]–[42]. Therefore past Caspian CDV epizootics may have caused periodic mortality peaks in the long term monitoring data, for instance a notable peak in spring mortality was recorded in 1971. We detected suggestive evidence for a 7 year period in spring mortality. The analysis has low power but a 7 year period is consistent with the epidemiology of morbilliviruses, [42], [43], and the observation may warrant further modelling.

There was a significant fall in carcases in the Apsheron monitoring zone since 2002, decreasing to one third the pre-1990 average. Rather than indicating a decrease in mortality, this may reflect decreased use of the waters around the peninsula due to disturbance from offshore oil infrastructure and industrial shipping, increased coastal urban development, and increases in fishing activity. From 2006, haul-out sites on the Apsheron peninsula, historically used by many 100 s to 1000 s of seals have been abandoned, despite the site being designated a National Park (T. Eybatov, unpublished data).

### Age determination and growth curves of Caspian seals

This study presented the first tooth growth layer based ageing together with body measurements of a large sample of Caspian seals. Asymptotic standard body length was estimated at approximately 126–129 cm. The only previous estimate of growth curves for Caspian seals was presented by McLaren [44], based on data on dorsal curvilinear lengths of 40 females from Chapskii [45]. McLaren estimated the female asymptotic standard length at approximately 10 years to be 133 cm.

In our study asymptotic lengths of males and females were compared for the first time, but were not significantly different. This may in part be due to the high standard deviation in body lengths of males. Females appear to reach their asymptotic length by 10 years of age, while male growth rates are lower, but growth may extend into the fourth decade of life. Due to the individual variability in growth and the early attainment of the asymptotic length, body length should not be used as an index of age of for post-juvenile seals. The growth pattern is similar to ringed seals (*Pusa hispida*), but differs from Baikal seals (*Pusa sibirica*), which do exhibit significant sexual dimorphism [25]. A lack of sexual dimorphism in size and secondary sexual characteristics is usually indicative of a lack of polygyny and inter-male competition during the breeding season, although sexual dimorphism may be ineffectual for seals that mate in the water [32], [46], [47]. Caspian seals have been observed towards the end of lactation to form pairs, with no evident inter-male competition (S. Wilson pers. obs).

### Age structure of Caspian seal mortality

Morbillivirus epizootics can generate significant age structure in mortality depending on relative exposure risks for different components of the population [40]–[42], [48]. The epidemiology of outbreaks will depend on the proportion of susceptible individuals, and timing of the epizootic relative to annual life-history. It would be expected that an epizootic of CDV would kill infected seals in all age groups which had not been previously exposed, and thus have no acquired immunity. The age distribution in 1997 is consistent with such a prediction [12], [39]. In subsequent epizootics the youngest age groups which have not been previously exposed, would be expected to suffer the highest death rates [41], [48], [49]. The observed age distribution of seal deaths in 2000 fits this prediction, with relatively high mortality for seals born in 1998–2000. Adults more than 22 years appear to be over-represented in 2000–2002. This could arise if they had increased susceptibility, increased exposure, or if there was bias in the stranding probability of carcases from this age group. Caspian seals form dense moulting aggregations in April in Komsomoletz Bay, Kazakhstan (Figure S6 in File S1), and Maly Zhemchuzhnyj Island, Russia, in which conditions are ideal for increased exposure to pathogens [41] via coughing, contact with mucus discharge, urine and faeces. Aggregations may be biased towards adult males, since most adult females may complete their moult on the ice-sheet [38]. This could create conditions for differential exposure rates, and therefore mortality, in different sex-age classes.

### Factors influencing organochlorine burdens in Caspian seals

We assessed potential contributions for Age, Blubber thickness and Sex to the variance in PCB and DDT burdens. All three variables explained significant variation when fitted singularly, but when fitted together, Age was strongly supported as accounting for most of the variation for both PCBs and DDTs, with levels of both positively correlated with Age in males and females. This fits with the general observations of OC accumulation over an individual’s lifetime seen in other marine mammals [50], [51]. Burdens were generally lower in females than males, although Sex was only a significant term in top ranked models for DDTs.

Transfer of OCs from mother seal to pup via lactation has long been established [52]. More recent work with grey seals has shown that the less lipophilic PCB-MSFs are most readily released into the mother’s milk, and thus passed to the suckling pup, which can excrete only about 0.5% of the contaminants [53]. A female’s first pup will therefore ingest the highest dose, but thereafter the levels should be lower. In a population bearing moderate POP burdens we might expect levels in young animals to be low, except for first-born pups [54]. OCs in juveniles <2 years old in our sample were low, with ∑PCBs ≤6 µg/g lipid weight and ∑DDTs≤13 µg/g lipid weight in 9 of 15 juveniles (Figure 6, Table S1 in File S1). OC concentrations in Caspian adult females were relatively low, with ∑PCBs ≤13 µg/g lipid weight and ∑DDTs≤35 µg/g lipid weight in 11 of 12 seals.

Blubber thickness is often cited as a confounding factor in studies of marine mammal toxicology, since apparent concentrations of contaminants may increase in blubber tissue as reserves are used up, and emaciated animals can appear to have higher burdens than individuals in good nutritive condition [9]. As reported by Kajiwara et al. [15] we also found a negative correlation between OC burden and blubber thickness, however, this source of variance appears to be less important than the contribution arising from Age. Blubber thickness appeared to account for ∼7, and ∼2.6 times less variance than Age in PCBs and DDTs respectively. Sample sizes in this study may limit power to fully resolve sources of variation in OC burdens, particularly for PCBs where the effect sizes may be smaller. This may also contribute to the observation of significant interaction terms for Blubber thickness in the linear models, without Blubber thickness itself being identified as a significant term.

### Levels of organochlorines in relation to CDV infections

The blubber concentrations of ∑PCBs and ∑DDTs were generally lower in CDV+ seals than in seals which apparently died of other causes, although the difference between the CDV+ and CDV− groups was not significant. The finding that CDV+ animals did not have relatively elevated PCB and DDT levels confirms the conclusion of Kuiken et al. [11] using data from spring 2000 alone. Among seals with the highest OC levels, of three adults with concentrations of ∑PCBs >100 µg/g lipid weight, only one was CDV+, and of five seals with concentrations of ∑DDTs >400 µg/g lipid weight, only one seal was CDV+ (Table S1 in File S1). ∑DDT concentrations in Caspian seal blubber are relatively high compared with seals in Europe and comparable to Baikal seals [15]. PCBs are lower than European seal populations, comparable to Baikal seals but higher than levels found in Arctic species [15].

A logistic regression analysis did not identify any variables making a significant contribution to variation between the CDV+ and CDV− groups. Again the analysis is likely to suffer from low power due to the small number of individuals falling in the CDV− category. Overall there is no support for the hypothesis that ∑DDTs or ∑PCBs were a significant factor in the Caspian CDV epizootic.

In these comparisons samples were categorised according to whether they were CDV+ or CDV− on the basis of RT-PCR or immunohistochemistry diagnostics. However, this should not be regarded as an infectious disease case control study since the CDV− negative animals contained individuals which might have died from other infectious diseases [11].

Environmental organochlorines occur in complex mixtures and there is relatively little information on which compounds present the highest risks for marine mammal health. The Caspian seal contaminant burdens included a mixture of DDTs and PCBs. Although DDT concentrations were higher than PCBs, DDTs are 3–4 orders of magnitude less toxic than PCBs [3]. Therefore PCB concentrations may be most relevant to immune impairment. A threshold for blubber PCB concentrations for the start of immune impairment in aquatic mammals was proposed at 17 µg/g lipid weight [3], and some evidence for this was found in harbour porpoises [9], [55]. The threshold for PCBs proposed by Kannan et al. [3] was exceeded in 29 (43%) of the 67 Caspian seals in our samples. This suggests that there could be some potential for immune impairment in individual Caspian seals, probably increasing with age, even though elevated PCB or DDT concentrations were not associated with CDV-induced mortality at the population level. Hall et al. [9] suggested that in harbour porpoises, for each 1 µg/g lipid weight increase in blubber PCBs, the risk of disease mortality increased by 2%, doubling at 45 µg/g lipid weight, but cautioned that sensitivity to OC toxicity can varying considerably among species, and general thresholds for impacts may need to be applied cautiously.

Ah-receptor mediated PCBs, PCDDs (dioxins) and PCDFs (furans), measured as TEQs, may have the greatest immunotoxic potential [1]. Kajiwara et al. [15] previously presented TEQ concentrations for 11 of the 67 Caspian seals for which OCs were analysed in this study. TEQs in the blubber of all animals investigated in spring 2000 were relatively low (10–340 pg/g lipid weight), and concentrations were lower in the 9 CDV+ seals (average 41 pg/g lipid weight) than the two CDV– adults (average 315 pg/g lipid weight; Tables 5, S1 in File S1).

**Table 5:** Comparison of Total TEQs in Caspian seals dying in 2000 with seals in the Netherlands feeding experiment (experimental data from Ross et al., 1995; Caspian data from Kajiwara et al. 2002; Kuiken et al. 2006).

	Experimental data		All Caspian deaths 1997−2002	
Total TEQ (pg/g lipid weight)	Exp	Control	CDV+	CDV−
**N**	11	11	21	9
**Mean age (Years)**	2–3	2–3	13	25
**Range (Years)**	–	–	<1–31	<1–33
**Mean TEQ (pg/g lipid weight)**	208	62	51	106
**Range (pg/g lipid weight)**	196–220	58–66	13–197	39–340

There was a strong correlation between log10(TEQs) and log10(PCBs) (adjusted *R* ^2^∶0.8134, *F*_1,10_∶48.96, *P* = 3.728e^−05^, so predicted TEQs were generated for the remaining seals via a linear model. These actual and predicted TEQ concentrations were compared to ∑TEQ levels observed in experiments with harbour seals [56]. Levels of 196−220 pg/g lipid weight and 58–66 pg/g lipid weight were seen for experimental and control groups of juvenile harbour seals which had been fed contaminated and uncontaminated fish respectively over a 126 week period [1], [56]–[62]. Impaired immune responses were found in the experimental group, and therefore ∑TEQ levels of around 200 pg/g lipid weight were considered to be immunotoxic, although Kannan et al. (2000) suggest a threshold of ∼520 pg/g lipid weight for marine mammals. Only 3 Caspian seals, one CDV+, one CDV−, one unclassified, had estimated TEQs within the immunotoxic range of the Dutch experiment (Figure 6). Therefore, overall there is nothing to suggest that ∑TEQ levels in Caspian seals facilitated or enhanced the severity of the CDV epizootic.

Organochlorine concentrations in several species of prey fish from different areas of the Caspian were analysed by Kajiwara et al. [15]. Comparing these with OC concentrations in the fish fed to the control and experimental groups of seals in the Netherlands experiment [1], [60], the ΣPCB and ΣDDT levels in the Caspian fish (11–108 and 5–184 µg/g lipid weight respectively) were similar to those of the relatively uncontaminated Atlantic fish fed to the Netherlands control group (260 and 102 µg/g lipid weight respectively) and very much less than those of the relatively contaminated Baltic fish (1460 and 497 µg/g lipid weight respectively) fed to the experimental group. Only the HCHs in some fish from Iran and Turkmenistan were in the range of the contaminated Baltic fish. From these data there is therefore no evidence that OC contaminants in Caspian fish presented a sufficiently high level of chronic exposure to seals to have an immunotoxic effect.

### Are OC levels in Caspian seals sufficiently high to impair fertility?

The pregnancy rate in Caspian seals in recent years has been reported to be as low as 30–35% [22], [38], [63]. This low rate has been attributed to assumed high OC burdens, by analogy with the relationship demonstrated in Baltic ringed seals between pathological changes in the uterus (stenosis and occlusions), and PCB and DDT levels [2], [24], [64].

The threshold PCB and DDT levels accumulated in the blubber associated with these pathological changes appear to be about 70 µg/g lipid weight ∑PCBs and 80 µg/g lipid weight ∑DDTs [2]. These PCB and DDT levels were rare among female Caspian seals in our sample, and were observed in only one female out of 13. Uterine pathologies and claw deformities as recorded in the Baltic have not been described in Caspian seals. ∑DDT levels in the adult male Caspian seals in our sample exceeded 100 µg/g lipid weight in 15/33 adult males, but the impact of such levels on male seal reproductive function is unknown.

### Seasonal variation in body condition in relation to toxicology, disease epidemiology and mortality

Seasonal variation in body condition is a normal feature of the annual life-history of Caspian and other phocid seal species, which reduce their foraging during breeding and moulting seasons, later rebuilding reserves with a period of intensive foraging [65], [66]. Blubber reserves in Caspian seals peak in the autumn-winter following summer foraging, and are at a minimum around April at the end of the moult, before they are restored. Our data shown that mean blubber thickness of seals dying in May-June of the epizootic years was significantly lower than in May-June of the more recent, non-epizootic years, but there was no significant difference in mean blubber thickness between CDV+ and CDV− animals. Linear models showed that blubber thickness explained only a relatively small proportion of variance in OC burdens compared to age.

Laboratory studies of seals during a fasting period found that some immune impairment did occur when seals have a negative energy budget, although this was independent of organic contaminant levels [58]. Reduced immune responsiveness and decreased survival associated with poor nutrient levels in other species is thought to be due to the additional energetic requirements of mounting an antigen-induced immune response [67]–[69]. The emaciation of carcases seen during epizootics in May-June was therefore likely to be a *consequence* of debilitation due to the immune response and impaired feeding due to illness, rather than the *cause* of immune suppression via increased OC levels in the organs. CDV infection itself causes severe immune suppression [70], which may swamp immune suppression due to elevated OC levels. The epidemiology of CDV provides an alternative driver for the high mortalities of adults recorded in May–June 1997 and 2000–01. Contact rates and transmission opportunities would be much higher during the annual moult compared to the rest of the year, while individuals are also undergoing physiological stress due to the moulting process and limited feeding.

### Impacts of CDV epizootic mortality on Caspian seal population status

The minimum current Caspian seal population size, estimated, from annual aerial surveys of pup production 2005–06, is ∼104,000 [16], [17], and Dmitrieva et al. [71] estimate the safe Potential Biological Removal (PBR) for the species at ∼3200 individuals per year. The actual total CDV epizootic mortality is not known, but by June 2000, 3654 carcasses had been recorded in Kazakhstan. Government and media sources around the Caspian region cited mortality as ∼10,000 seals in 2000, although the basis for this figure is not clear [11]. Caspian-wide mortality in 1997 and 2001 may have been lower, although there was more limited media coverage and fewer organised carcase counts in these years.

Despite the uncertainty over total epizootic mortality, epizootics have the potential to exceed the population PBR, and therefore recurrent CDV epizootics could contribute to a population decline. However, due to the infrequent occurrence of epizootics, anthropogenic sources of mortality are likely to be more important drivers of decline. Dmitrieva et al. [71] showed that by-catch in illegal sturgeon fisheries is likely to exceed the PBR on an annual basis, and a hind-casting reconstruction of Caspian seal demographic history from hunting records suggests that hunting alone can account for the historical population decline [16].

The origin of the epizootic CDV strain in Caspian seals is still unknown, with no exact match to known strains in either pinnipeds or terrestrial carnivores Kuiken et al. [11]. It remains to be determined if the current seal population size and contact rates are sufficient to maintain endemic circulation of CDV, or whether future CDV epizootics would require introduction of the virus from a terrestrial reservoir such as dogs, jackals or wolves. Virological surveys of canids around the Caspian should be a priority to determine if such species were indeed the historical source and could act as reservoirs for future outbreaks.

The current study suggests that there is little evidence that OC burdens were a significant factor in the CDV epizootics, and therefore priorities for conservation of Caspian seals would be more productively focused on reducing human caused mortality and protecting key habitats. However, given the elapsed time since the last evaluation of OC burdens in Caspian seals, the ongoing urbanisation and industrialisation around the Caspian Sea, and the need to determine OC toxicity thresholds for individual species, further research on contaminants and health in Caspian seals is important for understanding the overall status of the population. Future work should aim to conduct formal infectious disease case-control studies, with larger and more balanced sampling than was possible in what was an emergency response to the 1997–2001 epizootics. This would require long-term stranding investigation programmes, and health studies with non-lethal sampling of live seals. However, at present none of the Caspian countries are conducting such research.

## Supporting Information

File S1 **Contains Figures S1–S6 and Tables S1–S5.** Figure S1. Map of study areas and sampling sites. Table S1. Pathology and toxicology data for necropsied Caspian seals 1997–2002. Table S2. Age, sex and body length data used to generate Caspian seal growth curves. Figure S2. Overlain data and growth curves for male (filled points, black line) and female (open triangles, grey line) Caspian seals. Table S3. Linear model comparison for PCBs ranked by AIC score. Table S4. Linear model comparison for DDTs ranked by AIC score. Figure S3. Boxplots showing variation in PCBs and DDTs for Country, Season and Year. Figure S4. Plots for analysis of blubber thickness. Figure S5. Plots for regression comparisons of PCBs and DDTs. Table S5. Results of general linear binomial model with logit link function, assessing contributions of organochlorine burden, blubber thickness and sex to CDV status. Figure S6. Photograph showing a high density moulting aggregation of Caspian seals in Komsomoletz Bay, Kazakhstan.
(PDF)
