# Supplementary material for: The Role of Canine Distemper Virus and Persistent Organic Pollutants in Mortality Patterns of Caspian Seals (Pusa caspica)
Source: PLoS One. 2014 Jul 2;9(7):e99265. doi: 10.1371/journal.pone.0099265 (PMC4079250; doi:10.1371/journal.pone.0099265)

Supplementary Information

Figure S1. Map of study areas and sampling sites. S2

Table S1. Pathology and toxicology data for necropsied Caspian seals 1997-2002. S3

Table S2. Age, sex and body length data used to generate Caspian seal growth curves. S6

Figure S2. Overlain data and growth curves for male (filled points, black line) and female (open triangles, grey line) Caspian seals. S9

Table S3. Linear model comparison for PCBs ranked by AIC score. S10

Table S4. Linear model comparison for DDTs ranked by AIC score. S11

Figure S3. Boxplots showing variation in PCBs and DDTs for Country, Season and Year. S12

Figure S4. Plots for analysis of blubber thickness. S13

Figure S5. Plots for regression comparisons of PCBs and DDTs. S14

Table S5. Results of general linear binomial model with logit link function, assessing contributions of organochlorine burden, blubber thickness and sex to CDV status. S15

Figure S6. Photograph showing a high density moulting aggregation of Caspian seals in Komsomoletz Bay, Kazakhstan. S16

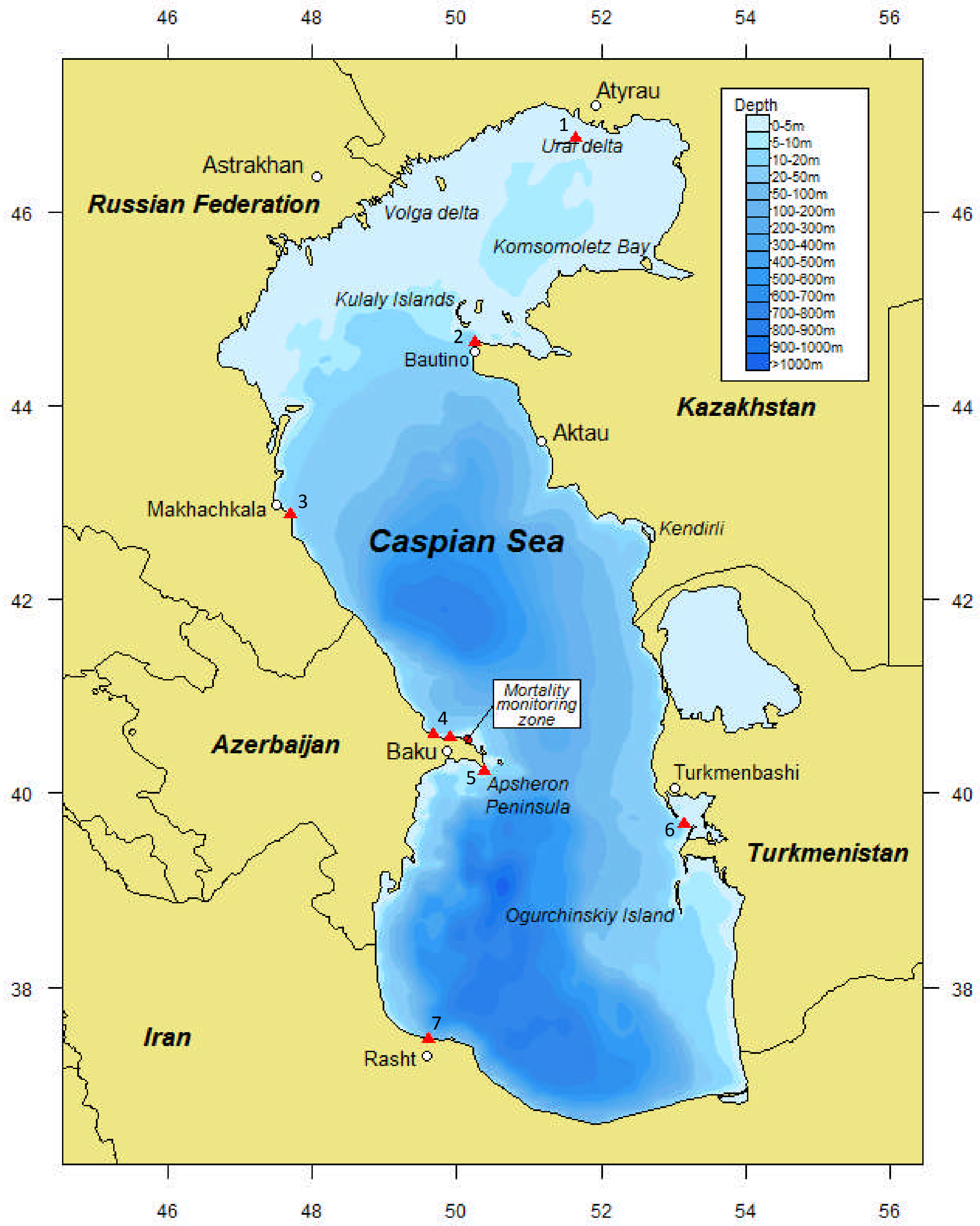

Figure S1. Map of study areas and sampling sites.

Map of the Caspian Sea showing surrounding countries, major cities, sampling locations for carcases (▲; 1: Zud West Island; 2: Bautino; 3: Dagestan; 4: Kurdakhany, Mardakyan, N. Apsheron, Pirshaga, Novkhany, Sumgait; 5: Shakhova Kosa; 6: Osushnoy, Geneje; 7: Guilan), Apsheron monitoring zone, and sea areas referred to in the text (italicised names). The map was generated by the authors in the R statistical package using open source data in the rworldmap package. (C) Simon Goodman, Creative Commons Attribution License.

Table S1. Pathology and toxicology data for necropsied Caspian seals 1997-2002.

NA indicates missing data.

Seal	Original.Seal.ID	Country	Location	Date	Season	Sex	Age	Age class	Body length (cm)	Blubber (cm)	IHC	RT. PCR	CDV status	Bacteria	Sum PCBs	Sum DDTs	TEQs
KZ001	Kuiken-03/KZPC-05	Kazakhstan	ZW.Island	2000-05-04	Spring	F	0.5	Juvenile	NA	NA	pos	NA	pos	pos	1.25	6.1	NA
KZ002	Kuiken-04/KZPC-06	Kazakhstan	ZW.Island	2000-05-04	Spring	F	0.5	Juvenile	NA	NA	pos	NA	pos	NA	1.5	5.24	NA
KZ003	Kuiken-05/KZPC-07	Kazakhstan	ZW.Island	2000-05-04	Spring	F	NA	Juvenile	NA	NA	pos	NA	pos	pos	4.2	4.4	NA
KZ005	KZPC-2000-08	Kazakhstan	Bautino	2000-05-07	Spring	F	0.5	Juvenile	87	1	NA	NA	NA	NA	8.9	30	44
KZ006	KZPC-2000-09	Kazakhstan	Bautino	2000-05-07	Spring	F	0.5	Juvenile	78	2	pos	NA	pos	pos	5.8	12	36
KZ007	KZPC-2000-10	Kazakhstan	Bautino	2000-05-07	Spring	F	NA	Adult	115	2	NA	NA	NA	NA	18	23	73
AZ001	Hall-515/97-01	Azerbaijan	Kurdakhany	1997-06-14	Spring	M	NA	Adult	110	1.2	neg	neg	neg	NA	13.77	425.7	NA
AZ002	Hall-516/97-02	Azerbaijan	Kurdakhany	1997-06-14	Spring	M	NA	Adult	122	1.2	neg	neg	neg	NA	19.08	684	NA
AZ003	Hall-517/97-03	Azerbaijan	Mardakyan	1997-06-15	Spring	F	0.5	Juvenile	89	2.6	neg	neg	neg	NA	1.12	6.39	NA
AZ004	Hall-518/97-04	Azerbaijan	Mardakyan	1997-06-15	Spring	F	NA	Adult	109	0.8	neg	pos	pos	NA	6.62	73.5	NA
AZ005	BakuE/Kuiken#07	Azerbaijan	N.Apsheron	2000-05-14	Spring	M	NA	Adult	125	10.0	NA	pos	pos	NA	24	130	40
AZ006	Baku25/Kuiken#08	Azerbaijan	Pirshaga	2000-06-02	Spring	M	16	Adult	105	8.0	NA	pos	pos	NA	27	67	44
AZ007	Baku32/Kuiken#09	Azerbaijan	Pirshaga	2000-06-02	Spring	M	23	Adult	110	9.0	pos	pos	pos	pos	35	120	58
AZ008	Baku1/Kuiken#10	Azerbaijan	Shakhova.Kosa	2000-06-04	Spring	M	27	Adult	112	7.0	neg	neg	neg	pos	320	470	300
AZ009	Baku2/Kuiken#11	Azerbaijan	N.Apsheron	2000-06-05	Spring	F	22	Adult	106	15	pos	NA	pos	pos	10	17	31
AZ010	Baku3/Kuiken#12	Azerbaijan	Shakhova.Kosa	2000-06-05	Spring	F	23	Adult	107	0.0	neg	neg	neg	pos	120	230	340
AZ011	Baku4/Kuiken#13	Azerbaijan	N.Apsheron	2000-06-05	Spring	F	1	Juvenile	83	19	pos	pos	pos	pos	4.1	13	21
AZ012	Baku5/Kuiken#14	Azerbaijan	Shakhova.Kosa	2000-06-05	Spring	F	0	Juvenile	80	21	pos	pos	pos	pos	2.4	6.3	10
AZ013	Baku6/Kuiken#15	Azerbaijan	N.Apsheron	2000-06-07	Spring	M	1	Juvenile	77	7.0	pos	neg	pos	pos	26	64	90
AZ014	AZ-00-#07	Azerbaijan	NA	2000-06-25	Spring	M	27	Adult	123	2.0	NA	NA	NA	NA	110	520	NA
AZ015	AZ-00-#22	Azerbaijan	NA	2000-07-30	Spring	M	1	Juvenile	90	2.0	NA	pos	pos	pos	2.7	7.3	NA
AZ016	AZ-01-#04	Azerbaijan	Novkhany	2001-06-22	Spring	M	29	Adult	132	1.0	NA	pos	pos	NA	50	210	NA
AZ017	AZ-01-#05	Azerbaijan	Novkhany	2001-06-22	Spring	F	31	Adult	123	1.2	NA	pos	pos	pos	24	56	NA
AZ018	AZ-01-#06	Azerbaijan	Novkhany	2001-06-22	Spring	M	26	Adult	132	1.5	NA	neg	neg	NA	23	120	NA

AZ019	AZ-01-#07	Azerbaijan	Novkhany	2001-06-22	Spring	F	8	Adult	117	2.5	NA	pos	pos	pos	4.0	10	NA
AZ020	AZ-01-#08	Azerbaijan	Sumgait	2001-06-22	Spring	M	28	Adult	132	2.0	NA	pos	pos	NA	21	110	NA
AZ021	AZ-01-#09	Azerbaijan	Novkhany	2001-06-23	Spring	F	NA	NA	122	1.5	NA	NA	NA	pos	13	34	NA
AZ022	AZ-01-#10	Azerbaijan	Novkhany	2001-06-23	Spring	F	10	Adult	124	2.0	NA	NA	NA	NA	4.8	10	NA
AZ023	AZ-01-#11	Azerbaijan	Novkhany	2001-06-23	Spring	M	28	Adult	122	1.5	NA	NA	NA	pos	36	110	NA
AZ024	AZ-01-#12	Azerbaijan	Novkhany	2001-06-23	Spring	M	27	Adult	132	1.0	NA	NA	NA	NA	39	170	NA
AZ025	AZ-01-#13	Azerbaijan	Novkhany	2001-06-23	Spring	M	11	Adult	121	1.5	NA	NA	NA	neg	30	89	NA
AZ026	AZ-01-#14	Azerbaijan	Novkhany	2001-06-23	Spring	M	29	Adult	130	2.0	NA	NA	NA	NA	24	74	NA
AZ027	AZ-01-#15	Azerbaijan	Novkhany	2001-06-23	Spring	M	28	Adult	121	0.8	NA	NA	NA	pos	46	200	NA
AZ028	AZ-01-#16	Azerbaijan	Shakhova.Kosa	2001-06-24	Spring	M	0.5	Juvenile	90	2.0	NA	pos	pos	NA	2.1	3.1	NA
AZ029	AZ-01-#17	Azerbaijan	Shakhova.Kosa	2001-06-24	Spring	M	0.5	Juvenile	87	2.0	NA	pos	pos	pos	4.0	95.0	NA
AZ030	AZ-01-#18	Azerbaijan	Shakhova.Kosa	2001-06-25	Spring	M	NA	Adult	108	NA	NA	pos	pos	pos	160	640	NA
AZ030	AZ-01-#19	Azerbaijan	Shakhova.Kosa	2001-06-25	Spring	M	NA	Adult	108	1.2	pos	NA	pos	pos	22	69	NA
AZ031	AZ-01-#20	Azerbaijan	Shakhova.Kosa	2001-06-25	Spring	M	NA	Adult	100	2.0	pos	NA	pos	pos	3.1	13	NA
AZ032	AZ-01-#21	Azerbaijan	Shakhova.Kosa	2001-06-25	Spring	M	NA	Adult	119	6.0	NA	pos	pos	pos	4.8	15	NA
AZ033	AZ-02-N1	Azerbaijan	Shakhova.Kosa	2002-03-26	Spring	F	NA	Juvenile	98	3.0	NA	pos	pos	NA	13	30	NA
AZ034	AZ-02-N2	Azerbaijan	Shakhova.Kosa	2002-03-26	Spring	M	0.5	Juvenile	81	3.0	NA	NA	NA	NA	1.3	8.5	NA
DG001	DagestanCS0002	Dagestan	Dagestan	2000-04-01	Spring	M	NA	NA	NA	NA	NA	NA	NA	NA	55	150	NA
DG002	DagestanCS0006	Dagestan	Dagestan	2000-04-01	Spring	M	NA	NA	NA	NA	NA	NA	NA	NA	150	430	NA
DG003	DagestanCS0011	Dagestan	Dagestan	2000-04-01	Spring	M	NA	NA	NA	NA	NA	NA	NA	NA	120	560	NA
DG004	DagestanCS0014	Dagestan	Dagestan	2000-04-01	Spring	F	NA	NA	NA	NA	NA	NA	NA	NA	57	140	NA
TK001	T1/Kuiken#16	Turkmenistan	Geneje	2000-06-10	Spring	M	0.5	Juvenile	89	1.2	NA	pos	pos	NA	4	95	13
TK002	T2	Turkmenistan	Osushnoy	2001-04-15	Spring	M	NA	Adult	117	1.5	neg	pos	pos	pos	32	160	NA
IR001	CS-Iran-A	Iran	Guilan	Oct-Nov 2000	Autumn	M	NA	Adult	NA	5.5	NA	neg	neg	NA	2.6	9.5	NA
IR002	CS-Iran-B	Iran	Guilan	Oct-Nov 2000	Autumn	M	18	Adult	NA	2.5	NA	neg	neg	NA	36	130	NA
IR003	CS-Iran-C	Iran	Guilan	Oct-Nov 2000	Autumn	F	21	Adult	NA	4.0	NA	pos	pos	NA	8.9	30	NA
IR004	CS-Iran-D	Iran	Guilan	Oct-Nov 2000	Autumn	M	22	Adult	NA	6.0	NA	pos	pos	NA	5.5	25	NA
IR005	CS-Iran-E	Iran	Guilan	Oct-Nov 2000	Autumn	M	33	Adult	NA	2.0	NA	neg	neg	NA	28	140	NA
IR006	CS-Iran-F	Iran	Guilan	Oct-Nov 2000	Autumn	F	16	Adult	NA	4.0	NA	pos	pos	NA	1.9	4.5	NA
IR007	CS-Iran-G	Iran	Guilan	Oct-Nov 2000	Autumn	M	24	Adult	NA	4.5	NA	neg	neg	NA	7.0	30	NA
IR008	CS-Iran-H	Iran	Guilan	Oct-Nov 2000	Autumn	M	19	Adult	NA	5.0	NA	NA	NA	NA	11	41	NA

IR009	CS-Iran-I	Iran	Guilan	Oct-Nov 2000	Autumn	F	NA	Adult	NA	6.5	NA	NA	NA	NA	1.5	4.9	NA
IR010	CS-Iran-J	Iran	Guilan	Oct-Nov 2000	Autumn	M	22	Adult	NA	5.5	NA	pos	pos	NA	11	68	NA
IR011	CS-Iran-K	Iran	Guilan	Oct-Nov 2000	Autumn	M	43	Adult	NA	7.0	NA	NA	NA	NA	2.8	6.7	NA
IR012	CS-Iran-L	Iran	Guilan	Oct-Nov 2000	Autumn	M	22	Adult	NA	5.5	NA	NA	NA	NA	2.1	5.2	NA
IR013	CS-Iran-M	Iran	Guilan	Oct-Nov 2000	Autumn	M	21	Adult	NA	5.5	NA	pos	pos	NA	17	55	NA
IR014	CS-Iran-N	Iran	Guilan	Oct-Nov 2000	Autumn	F	NA	Adult	NA	5.0	NA	NA	NA	NA	3.2	5.8	NA
IR015	CS-Iran-O	Iran	Guilan	Oct-Nov 2000	Autumn	M	NA	Adult	NA	6.5	NA	NA	NA	NA	15	43	NA
IR016	CS-Iran-P	Iran	Guilan	Oct-Nov 2000	Autumn	F	28	Adult	NA	1.5	NA	NA	NA	NA	11	31	NA
IR017	CS-Iran-Q	Iran	Guilan	Oct-Nov 2000	Autumn	M	13	Adult	NA	3.5	NA	pos	pos	NA	9.5	34	NA
IR018	CS-Iran-R	Iran	Guilan	Oct-Nov 2000	Autumn	M	22	Adult	NA	4.5	NA	pos	pos	NA	18	55	NA
IR019	CS-Iran-S	Iran	Guilan	Oct-Nov 2000	Autumn	M	29	Adult	NA	4.0	NA	NA	NA	NA	6.6	30	NA
IR020	CS-Iran-T	Iran	Guilan	Oct-Nov 2000	Autumn	M	28	Adult	NA	2.0	NA	NA	NA	NA	13	33	NA

Table S2. Age, sex and body length data used to generate Caspian seal growth curves.

Sex	Age (Years)	Standard Body Length (cm)
F	0.5	78
F	22	106
F	23	119
F	1	83
F	0.5	80
F	31	123
F	8	117
F	10	124
F	35	126
F	26	124
F	20	139
F	27	132
F	5	128
F	3.5	118
F	23.5	116
F	8.5	128
F	32	136
F	25	135
F	27	121
F	17	130
F	15	120
F	22	132
F	9.5	137
F	9.5	123
F	8.5	130
F	19.5	122
F	5.5	127
F	6	113
F	1.5	108
F	42	130
F	23	128
F	0.5	88
F	12	129
F	2.5	103
F	9.5	111
F	30	129
F	8	125
F	35	121
F	35	123
F	30	129
F	0.7	83
F	20	133

F	12	120
F	14	113
F	23	121
F	15	126
F	6	114
F	19	128
F	12	126
F	22	131
F	19	131
F	22	131
F	26	140
F	29	131
F	10	125
F	10	131
F	22	132
F	0.8	93
F	6	116
F	15	135
F	36	120
F	8	118
F	0.8	82
F	8	117
F	12	124
F	13	118
M	16	105
M	23	110
M	27	112
M	1	77
M	1	96
M	0.5	89
M	29	132
M	26	132
M	28	132
M	28	122
M	27	132
M	11	121
M	29	130
M	28	121
M	0.5	90
M	0.5	97
M	31	126
M	24	134
M	1.5	105
M	0.5	81
M	3.5	104
M	33	140

M	21	133
M	8.5	116
M	0.5	93.5
M	0.5	101
M	4.5	112
M	10	100
M	34	127
M	13	135
M	20	133
M	3	105
M	3	114
M	24	120
M	2.8	104
M	0.8	94
M	17	131
M	0.8	94
M	1.8	110
M	0.8	74
M	26	131
M	16	127
M	27	118
M	32	134
M	10	97

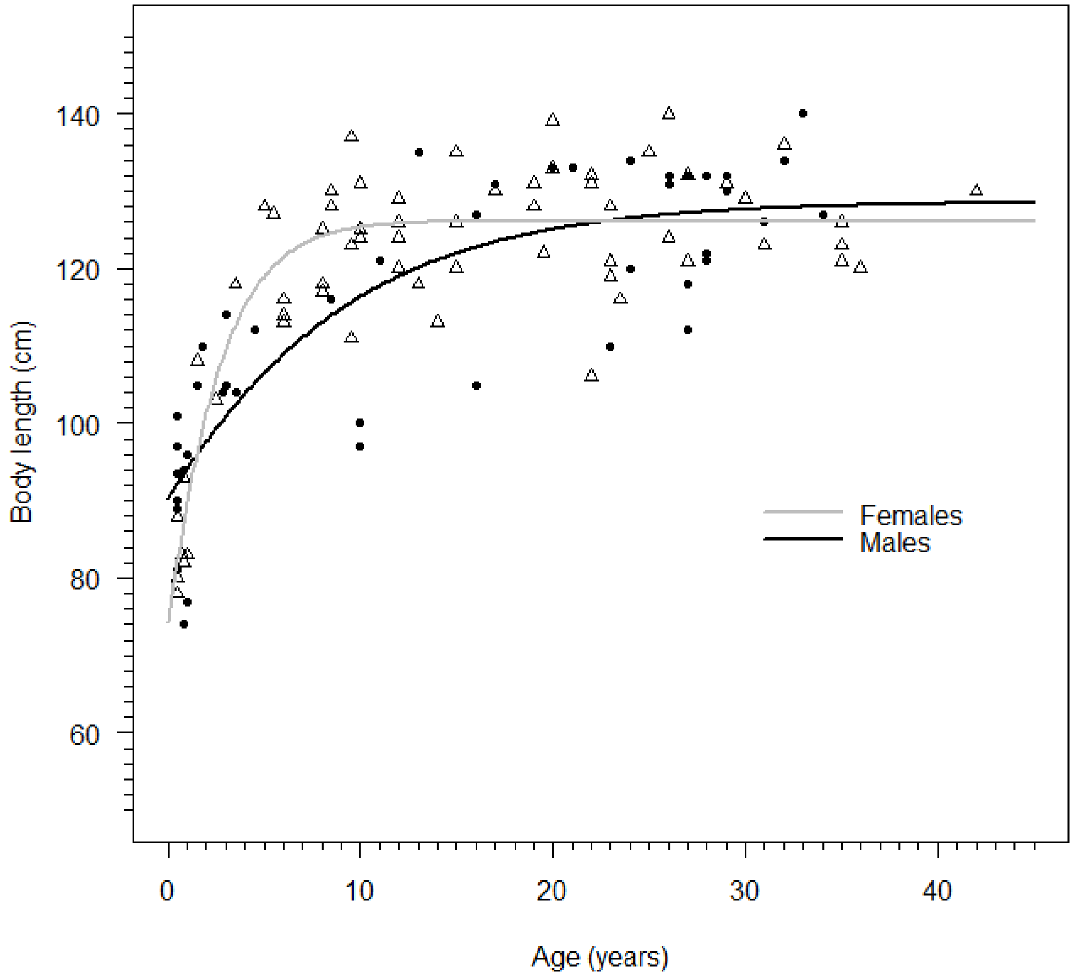

Figure S2. Overlain data and growth curves for male (filled points, black line) and female (open triangles, grey line) Caspian seals.

Table S3. Linear model comparison for PCBs ranked by AIC score.

Rank	Model	Adjusted R^2	*F* (df)	*p*-value	AIC	AIC df	ΔAIC	Evidence ratio
1	log10(PCBs)~Age+Age:log10(Blubber+1)+Sex:log10(Blubber+1)+Sex	0.374	7.408 (4,39)	0.00015	58.502	6	0.000	1.000
2	log10(PCBs)~Age+Age:log10(Blubber + 1))	0.339	12.03 (2,41)	0.00008	59.055	4	0.552	1.318
3	log10(PCBs)~Age+Age:log10(Blubber+1)+Sex	0.350	8.728 (3,40)	0.00014	59.216	5	0.713	1.429
4	log10(PCBs)~Age+log10(Blubber+1)+Age:log10(Blubber+1)+Sex:log10(Blubber+1)+Sex	0.358	5.801 (5,38)	0.00045	60.416	7	1.914	2.604
5	log10(PCBs)~Age+log10(Blubber+1)+Age:log10(Blubber+1)+Sex	0.339	6.518 (4,39)	0.00041	60.845	6	2.343	3.227
6	log10(PCBs)~Age*log10(Blubber+1)	0.324	7.882 (3,40)	0.00030	60.936	5	2.434	3.376
7	log10(PCBs)~Age*log10(Blubber+1)*Sex	0.367	4.554 (7,36)	0.00101	61.467	9	2.965	4.404
8	log10(PCBs)~Age+log10(Blubber+1)	0.292	9.866 (2,41)	0.00032	62.085	4	3.582	5.996
9	log10(PCBs)~Age+log10(Blubber+1)+Sex	0.290	6.848 (3,40)	0.00079	63.134	5	4.632	10.133
10	log10(PCBs)~Age+log10(Blubber+1)+Sex:log10(Blubber+1)+Sex	0.274	5.052 (4,39)	0.00223	65.000	6	6.498	25.763
11	log10(PCBs)~Age	0.321	22.28 (1,44)	0.00002	65.163	3	6.660	27.940
12	log10(PCBs)~Age+Sex	0.329	12.05 (2,43)	0.00007	65.538	4	7.036	33.716
13	log10(PCBs)~Age*Sex	0.325	8.205 (3,42)	0.00021	66.789	5	8.286	63.003
14	log10(PCBs)~log10(Blubber+1)	0.058	4.544 (1,57	0.03736	90.157	3	31.655	7.48E+06
15	log10(PCBs)~Sex	0.105	8.761 (1,65)	0.00429	113.037	3	54.534	6.95E+11

Table S4. Linear model comparison for DDTs ranked by AIC score.

Rank	Model	Adjusted R^2	F (df)	p-value	AIC	AIC df	ΔAIC	Evidence ratio
1	log10(DDTs)~Age+Age:log10(Blubber+1)+Sex)	0.3898	10.16 (3,40)	0.00004	58.755	5	0.000	1.000
2	log10(DDTs)~Age+Age:log10(Blubber+1)+Sex:log10(Blubber+1)+Sex)	0.381	7.616 (4,39)	0.00012	60.272	6	1.518	2.136
3	log10(DDTs)~Age+log10(Blubber+1)+Age:log10(Blubber+1)+Sex)	0.3743	7.430 (4,39)	0.00015	60.745	6	1.990	2.705
4	log10(DDTs)~Age+log10(Blubber+1)+Age:log10(Blubber+1)+Sex:log10(Blubber+1)+Sex)	0.3647	5.937 (5,38)	0.00038	62.271	7	3.516	5.800
5	log10(DDTs)~Age+log10(Blubber+1)+Sex)	0.3254	7.914 (3,40)	0.00029	63.167	5	4.413	9.082
6	log10(DDTs)~Age+Age:log10(Blubber+1))	0.2879	9.694 (2,41)	0.00036	64.633	4	5.878	18.898
7	log10(DDTs)~Age*log10(Blubber+1)*Sex)	0.3483	4.283 (7,36)	0.00156	65.014	9	6.259	22.866
8	log10(DDTs)~Age+log10(Blubber+1)+Sex:log10(Blubber+1)+Sex)	0.3102	5.835 (4,39)	0.00088	65.032	6	6.277	23.074
9	log10(DDTs)~Age*log10(Blubber+1))	0.2727	6.375 (3,40)	0.00124	66.477	5	7.722	47.516
10	log10(DDTs)~Age+log10(Blubber+1))	0.2573	8.448 (2,41)	0.00085	66.487	4	7.732	47.761
11	log10(DDTs)~Age+Sex)	0.3145	11.320 (2,43)	0.00011	67.938	4	9.183	98.653
12	log10(DDTs)~Age*Sex)	0.3088	7.700 (3,42)	0.00033	69.237	5	10.482	188.897
13	log10(DDTs)~Age)	0.235	14.820 (1,44)	0.00038	72.042	3	13.287	767.782
14	log10(PCBs)~log10(Blubber+1)	0.1312	9.760 (1,57)	0.00280	98.767	3	40.012	4.88E+08
15	log10(PCBs)~Sex	0.2079	18.330 (1,65)	0.00006	116.710	3	57.956	3.85E+12

Figure S3. Boxplots showing variation in PCBs and DDTs for Country, Season and Year.

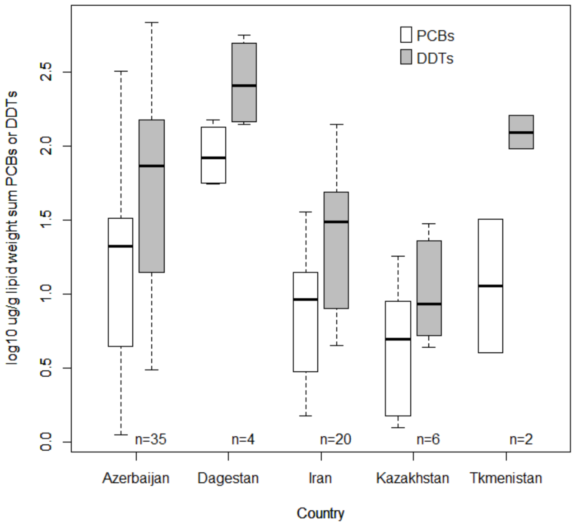

Figure S3a. Boxplot of Log10 PCBs and DDTs plotted against country.

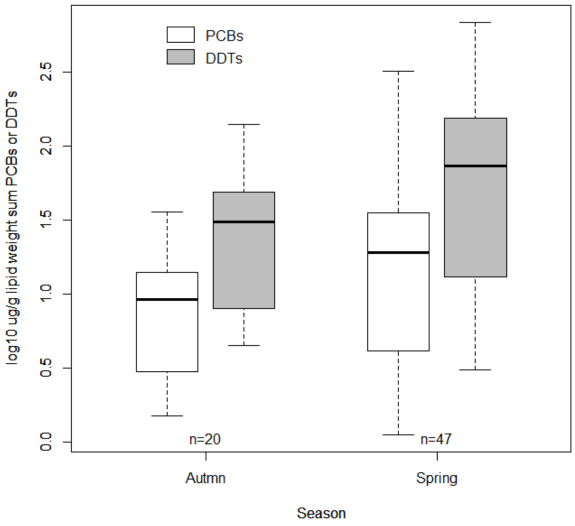

Figure S3b. Boxplot of Log10 PCBs and DDTs plotted against Season.

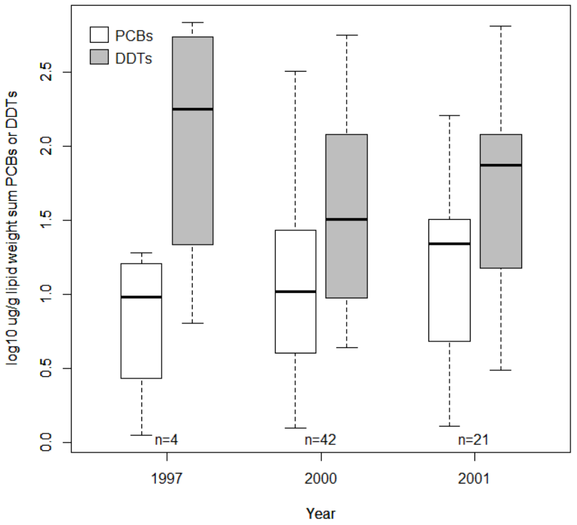

Figure S3c. Boxplot of Log10 PCBs and DDTs plotted against Year.

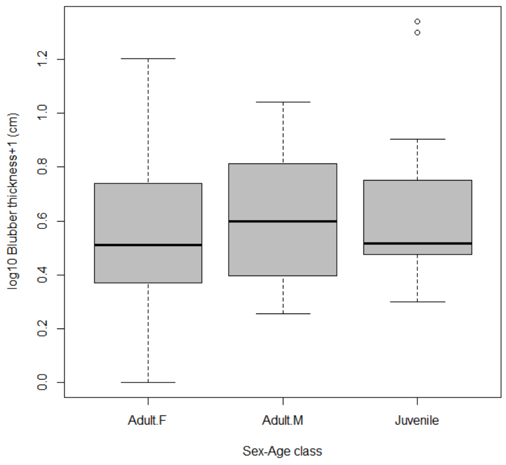

Figure S4a. Boxplot of log10(Blubber thickness+1) plotted against Sex-age class for samples included in toxicology analysis.

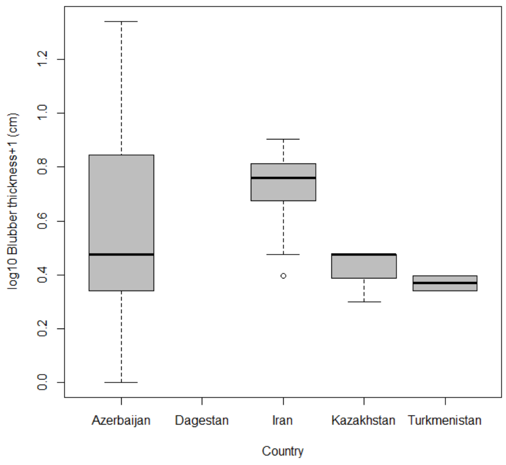

Figure S4b. Boxplot of log10(Blubber thickness+1) plotted against Country for samples included in toxicology analysis.

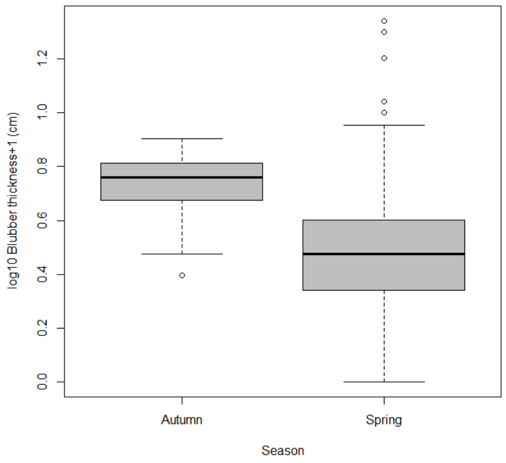

Figure S4c. Boxplot of log10(Blubber thickness+1) plotted against Season for samples included in toxicology analysis.

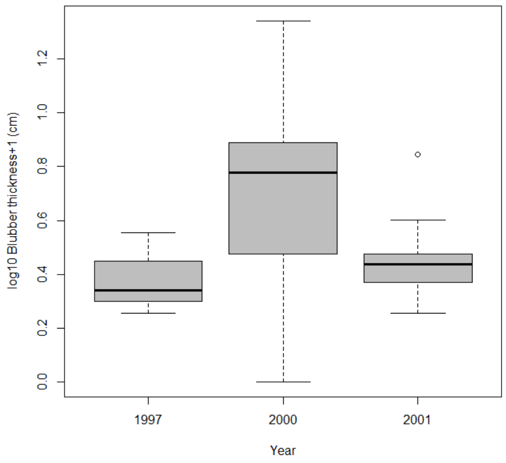

Figure S4d. Boxplot of log10(Blubber thickness+1) plotted against Year for samples included in toxicology analysis.

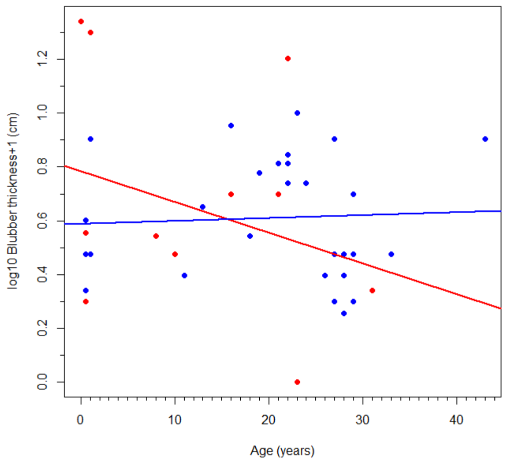

S4e. Log10 Blubber+1 (cm) against Age (years), with fitted values from linear regression for males (blue line, blue points) and females (red line, red points).

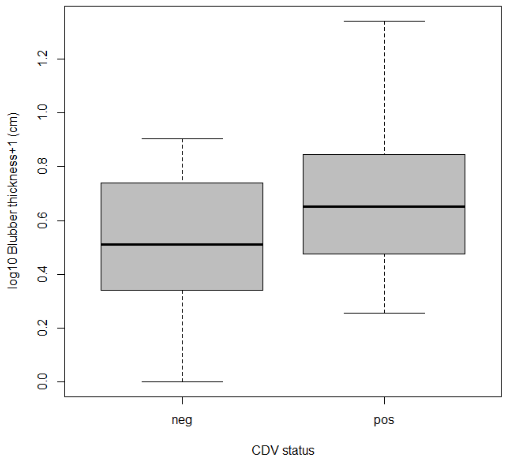

Figure S4c. Boxplot of log10(Blubber thickness+1) plotted against CDV status.

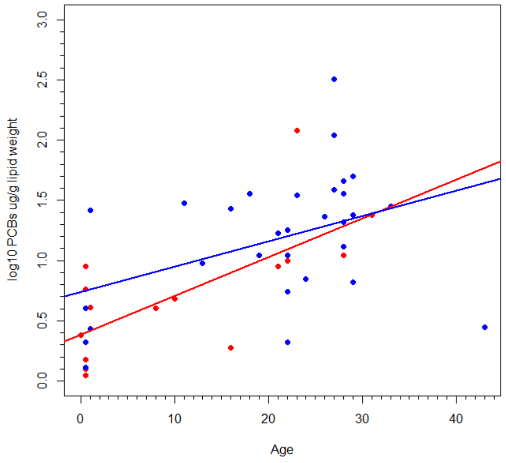

S5a. Log10 ∑PCB concentration against age (years), with fitted values from linear regression for males (blue line, blue points) and females (red line, red points).

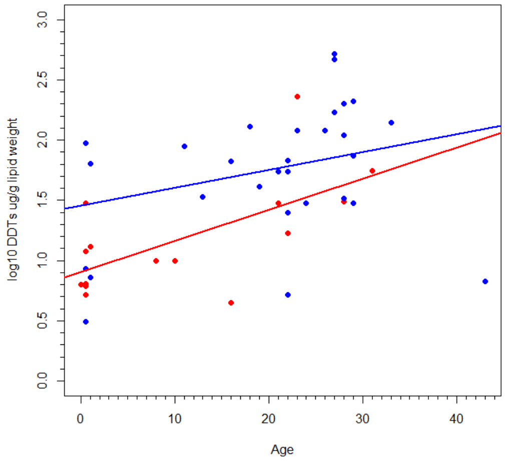

S5b. Log10 ∑DDT concentration against age (years), with fitted values from linear regression for males (blue line, blue points) and females (red line, red points).

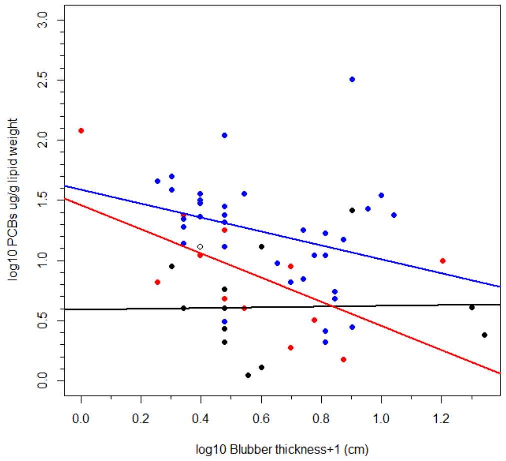

S5d. Log10 ∑PCB concentration against Log10 Blubber thickness+1 (cm), with fitted values from linear regression for adult males (blue line, blue points), adult females (red line, red points), and juveniles (black line, black points). Open point – age class not determined.

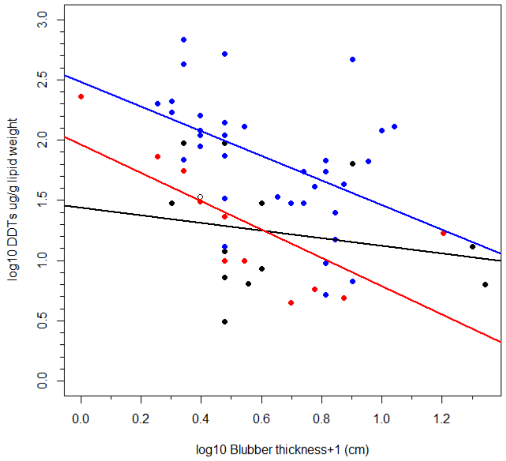

S5e. Log10 ∑DDT concentration against Log10 Blubber thickness+1 (cm), with fitted values from linear regression for adult males (blue line, blue points), adult females (red line, red points), and juveniles (black line, black points). Open point – age class not determined.

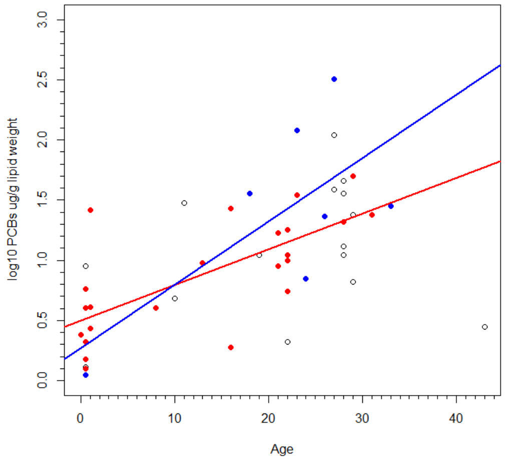

S5e. Log10 ∑PCB concentration against age (years), with fitted values from linear regression for CDV negative animals (blue) and CDV positive animals (red). Open points are animals with undetermined CDV status.

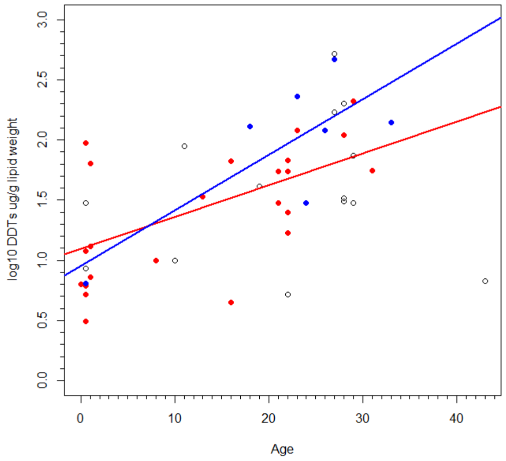

S5f. Log10 ∑DDT concentration against age (years), with fitted values from linear regression for CDV negative animals (blue) and CDV positive animals (red). Open points are animals with undetermined CDV status.

Table S5. Results of general linear binomial model with logit link function, assessing contributions of organochlorine burden, blubber thickness and sex to CDV status.

PCBs

CDV ~ log10(PCBs) + log10(Blubber + 1) + Age + Sex, family = binomial

```
Deviance Residuals:
    Min       1Q   Median       3Q      Max
-2.3999   0.1591   0.5165   0.6226   1.4407

Coefficients:
                   Estimate Std. Error z value Pr(>|z|)
(Intercept)         1.53826    1.79798   0.856    0.392
log10(PCBs)        -1.16556    1.21458  -0.960    0.337
log10(Blubber + 1)  2.43485    1.99854   1.218    0.223
Age                -0.02679    0.06157  -0.435    0.663
SexM               -0.11450    1.20511  -0.095    0.924

(Dispersion parameter for binomial family taken to be 1)

    Null deviance: 32.055  on 28  degrees of freedom
Residual deviance: 25.981  on 24  degrees of freedom
  (40 observations deleted due to missingness)
AIC: 35.981

Number of Fisher Scoring iterations: 5
```

DDTs

CDV ~ log10(DDTs) + log10(Blubber + 1) + Age + Sex, family = binomial

```
Deviance Residuals:
    Min       1Q   Median       3Q      Max
-2.3095   0.1793   0.4812   0.6896   1.4102

Coefficients:
                   Estimate Std. Error z value Pr(>|z|)
(Intercept)         2.56760    2.58063   0.995    0.320
log10(DDTs)        -1.26373    1.44885  -0.872    0.383
log10(Blubber + 1)  1.91100    2.03514   0.939    0.348
Age                -0.03577    0.05586  -0.640    0.522
SexM                0.29670    1.36528   0.217    0.828

(Dispersion parameter for binomial family taken to be 1)

    Null deviance: 32.055  on 28  degrees of freedom
Residual deviance: 26.100  on 24  degrees of freedom
  (40 observations deleted due to missingness)
AIC: 36.1

Number of Fisher Scoring iterations: 5
```

Figure S6. Photograph showing a high density moulting aggregation of Caspian seals in Komsomoletz Bay, Kazakhstan (Photo © Victoria Kovshar, used with permission under a Creative Commons Attribution License).

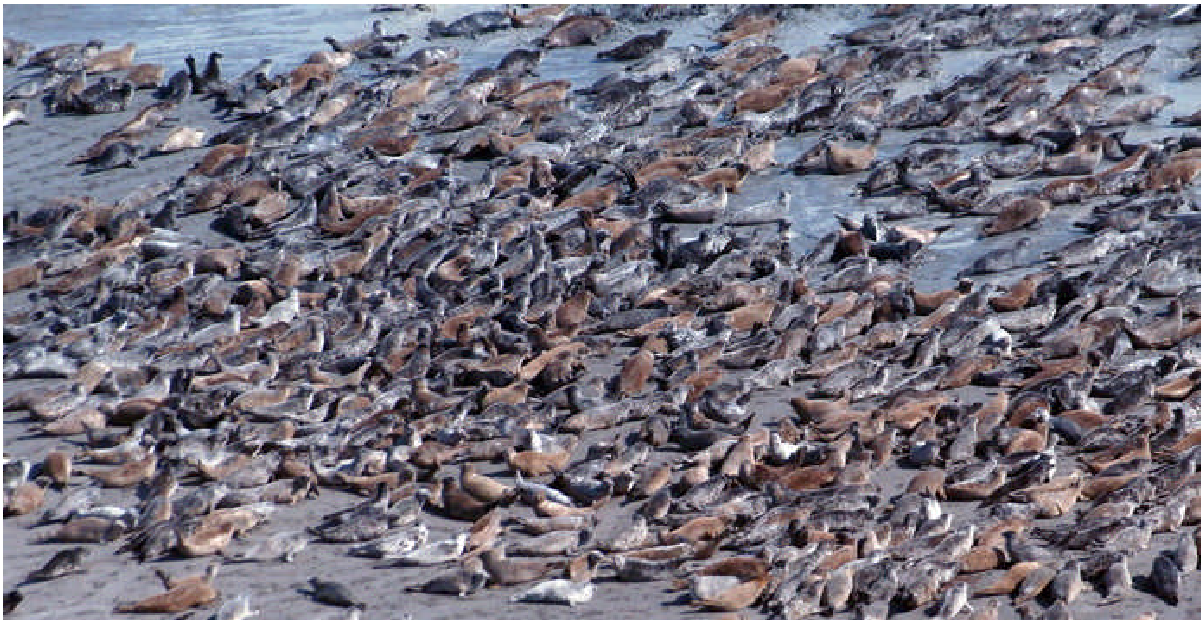

Supplement: File S1 — Contains Figures S1–S6 and Tables S1–S5. Figure S1. Map of study areas and sampling sites. Table S1. Pathology and toxicology data for necropsied Caspian seals 1997–2002. Table S2. Age, sex and body length data used to generate Caspian seal growth curves. Figure S2. Overlain data and growth curves for male (filled points, black line) and female (open triangles, grey line) Caspian seals. Table S3. Linear model comparison for PCBs ranked by AIC score. Table S4. Linear model comparison for DDTs ranked by AIC score. Figure S3. Boxplots showing variation in PCBs and DDTs for Country, Season and Year. Figure S4. Plots for analysis of blubber thickness. Figure S5. Plots for regression comparisons of PCBs and DDTs. Table S5. Results of general linear binomial model with logit link function, assessing contributions of organochlorine burden, blubber thickness and sex to CDV status. Figure S6. Photograph showing a high density moulting aggregation of Caspian seals in Komsomoletz Bay, Kazakhstan. (PDF) [file pone.0099265.s001.pdf]
